# A magnetic anti-cancer compound for magnet-guided delivery and magnetic resonance imaging

**DOI:** 10.1038/srep09194

**Published:** 2015-03-17

**Authors:** Haruki Eguchi, Masanari Umemura, Reiko Kurotani, Hidenobu Fukumura, Itaru Sato, Jeong-Hwan Kim, Yujiro Hoshino, Jin Lee, Naoyuki Amemiya, Motohiko Sato, Kunio Hirata, David J. Singh, Takatsugu Masuda, Masahiro Yamamoto, Tsutomu Urano, Keiichiro Yoshida, Katsumi Tanigaki, Masaki Yamamoto, Mamoru Sato, Seiichi Inoue, Ichio Aoki, Yoshihiro Ishikawa

**Affiliations:** 1 Cardiovascular Research Institute, Yokohama City University, Graduate School of Medicine, Yokohama, Japan; 2 Advanced Applied Science Department, Research Laboratory, IHI Corporation, Yokohama, Japan; 3 Biochemical Engineering, Faculty of Engineering, Yamagata University, Yonezawa, Yamagata, Japan; 4 Department of orthopedics, Machida Hospital, Machida, Tokyo; 5 Department of Oral Surgery, Yokohama City University, Graduate School of Medicine, Yokohama, Japan; 6 Nanoparticles by Design Unit, Okinawa Institute of Science and Technology Graduate University, Onna-son, Japan; 7 Department of Environment and Natural Sciences, Graduate School of Environment and Information Sciences, Yokohama National University, Yokohama, Japan; 8 Department of Radiology, Yokohama City University, Graduate School of Medicine, Yokohama, Japan; 9 Department of Electrical Engineering, Graduate School of Engineering, Kyoto University, Kyoto, Japan; 10 Department of Physiology, Aichi Medical University, Nagakute, Aichi, Japan; 11 RIKEN, Research Infrastructure Group, SR Life Science Instrumentation Unit, Hyogo, Japan; 12 Materials Science and Technology Division, Oak Ridge National Laboratory, TN, USA; 13 Tokyo Neutron Science Laboratory, Institute for Solid State Physics, the University of Tokyo, Shirakata, Tokai, Japan; 14 Department of Chemistry of Functional Molecules, Faculty of Science and Engineering, Konan University, Kobe, Japan; 15 Yokohama City University, Graduate School, Yokohama, Japan; 16 Department of Histology and Cell Biology, Yokohama City University School of Medicine, Yokohama, Japan; 17 WPI-AIMR & Department of Physics, Graduate School of Science, Tohoku University, Sendai, Japan; 18 Structural Biology Laboratory, Graduate School of Medical Life Science, Yokohama City University, Yokohama, Japan; 19 Molecular Imaging Center, National Institute of Radiological Sciences, Chiba, Japan

## Abstract

Research on controlled drug delivery for cancer chemotherapy has focused mainly on ways to deliver existing anti-cancer drug compounds to specified targets, e.g., by conjugating them with magnetic particles or encapsulating them in micelles. Here, we show that an iron-salen, i.e., μ-oxo *N*,*N'*- bis(salicylidene)ethylenediamine iron (Fe(Salen)), but not other metal salen derivatives, intrinsically exhibits both magnetic character and anti-cancer activity. X-Ray crystallographic analysis and first principles calculations based on the measured structure support this. It promoted apoptosis of various cancer cell lines, likely, via production of reactive oxygen species. In mouse leg tumor and tail melanoma models, Fe(Salen) delivery with magnet caused a robust decrease in tumor size, and the accumulation of Fe(Salen) was visualized by magnetic resonance imaging. Fe(Salen) is an anti-cancer compound with magnetic property, which is suitable for drug delivery and imaging. We believe such magnetic anti-cancer drugs have the potential to greatly advance cancer chemotherapy for new theranostics and drug-delivery strategies.

Molecularly targeted drugs, such as antibody-based drugs, are known to bind to specific molecules in tissues. However, once they are administered, their target can no longer be changed. In contrast, magnetically based systems may have the advantage that delivery can be guided with magnet, even after drug administration, simply by applying a magnetic field. Because drugs are not intrinsically magnetic, various approaches have been investigated^1^,^2^. For example, superparamagnetic particles have been combined with drugs^3^,^4^ via ionic conjugation or by emulsification in micelles^5^. Their usefulness has been proposed for a broad range of biological applications, including cancer chemotherapy, tissue repair, hyperthermic therapy, or magnetic resonance (MR) imaging contrast enhancement^6^. However, such systems have rarely been applied clinically because of their intrinsic limitations; ionic conjugation may be readily broken, and micelles can be heat-denatured or lost during systemic circulation *in vivo*^7^. If drug compounds were inherently magnetic, most, if not all, of these problems might be solved.

*N*,*N*′-Bis(salicylidene)ethylenediamine, or salen, was first developed as a ligand in organometallic chemistry in 1969^8^, and the magnetic property of such metal salen derivatives, such as hydroxy-salicylidene-ethylendiamine-iron complexes^9^,^10^, has repeatedly examined because of its unique salen structure. Unfortunately, however, these salen iron complexes do not have sufficient magnetic property for drug delivery even if it could show magnetic behavior under specific conditions^9^. More recently, it was found that some of these metal salen derivatives exhibit cytotoxicity^11^,^12^, as exemplified by chloro(*N*,*N*′-ethylenebis(salicylideneaminato))iron ([Fe(salen)]Cl)^13^,^14^. Accordingly, these metal salen derivatives are now considered as potential anti-cancer compound.

It is very labor intensive to examine the presence of magnetisms of numerous metal salen derivatives by experiments. However, magnetism could be assessed with chemical structure *in silico* by the first principles calculations, a numerical simulation used in heavy industrial fields^15^. Here, we describe a previously un-investigated salen iron derivative, i.e., μ-oxo *N*,*N*′- bis(salicylidene)ethylenediamine iron (Fe(Salen)), that shows both cytotoxicity and magnetization. We demonstrate that such properties can be used, in an animal cancer model, for magnet-guided drug delivery and visualization of the accumulated drug by MR imaging.

## Results

### Anti-cancer property

**Figure 1:**
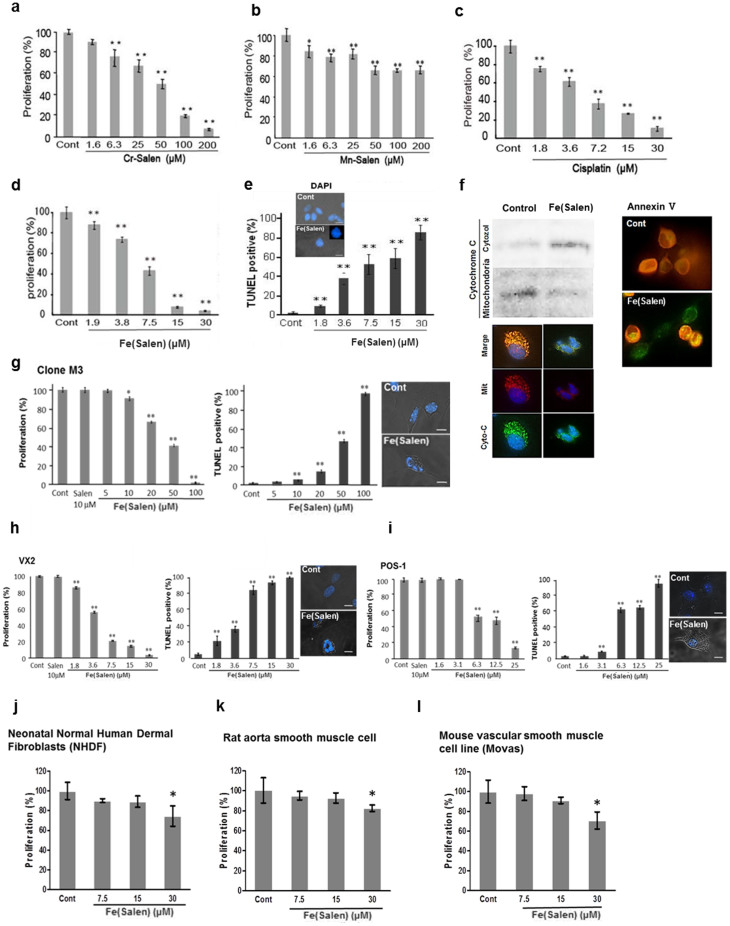
Anti-cancer effects of salen compounds in cultured cells. (a–f), Effect of various salen compounds on rat prostatic adenocarcinoma cells (MAT-Lu) cells. Cells were cultured in the presence of various salen compounds and analyzed by XTT assays: Cr-salen (a), Mn-salen (b), cisplatin (c), and Fe(Salen) (d). (n = 4, *p < 0.05, **p < 0.01 vs. control). The induction of apoptosis, evaluated by TUNEL assay, is also shown for Fe(Salen) (n = 10, **p < 0.01 vs. control) (e). DAPI staining for nuclei of non-treated (*Cont*) and Fe(Salen)-treated cells are shown. Immuno-histochemical staining for apoptosis (f). *Left*; Cytochrome *c* localization was compared between cytoplasm- and mitochondria-enriched fractions by immunoblotting in the presence or absence of Fe(Salen). Immunohistochemical staining of mitochondria (*red*) and cytochrome c (*green*) is also shown; co-localization is indicated by yellow in the merged images. Note that cytochrome *c* was released into the cytoplasmic fraction in the presence of Fe(Salen). *Right*; Annexin-V binding to the cell surface (*green*). Note that Annexin-V bound to cell membranes only in the presence of Fe(Salen), suggesting induction of apoptosis. (g–l), Effect of Fe(Salen) on various cancer and normal cells. Similarly, cell proliferation was assessed by XTT assays (n = 4, *p < 0.05, **p < 0.01 vs. control), and apoptosis by TUNEL assays (n = 10, **p < 0.01 vs. control). DAPI staining of cancer cells is also shown. The IC_50_ values were approximately 22 μM (clone M3 melanoma cells (g), 3 μM (VX2 rabbit squamous cell carcinoma (h), and 11.5 μM (POS-1 mouse osteosarcoma (i). Fe(Salen) exhibited significant but lower cytotoxicity towards non-cancer cells, such as human dermal fibroblast cells (NHDF, primary, j), rat aortic smooth muscle cells (primary, k), and mouse vascular smooth muscle cell lines (Movas, l).

As reported previously^11^,^12^, we confirmed that some metal salen derivatives, such as *N*,*N*′-bis(salicylidene)ethylenediamine chromium (Cr-salen) but not *N*,*N*′-bis(salicylidene)ethylenediamine manganese (Mn-salen), exhibited potent anti-cancer properties alike cisplatin (Fig. 1a, b and c). We also examined a previously unexamined iron salen, i.e., the commercially available *N*,*N*′-bis(salicylidene)ethylenediamine iron particles (Tokyo Chemical Industry (TCI product code D2571), and found that it also exhibited similar anti-cancer properties (Fig. 1d) and induced cellular apoptosis with similar efficacy to cisplatin, a widely used anti-cancer drug, in MAT-Lu prostate cancer cells (Fig. 1e and f). However, its apoptosis-inducing efficacy varied significantly among other cell types, such as melanoma (Clone M3), squamous cell carcinoma (VX2), osteosarcoma cells (POS-1) (Fig. 1g, h, and i). It also exhibited anti-proliferation effect, but to a lesser degree, on non-cancer cells, such as fibroblasts, and rat and mouse smooth muscle cell (Fig. 1j–l).

**Figure 2:**
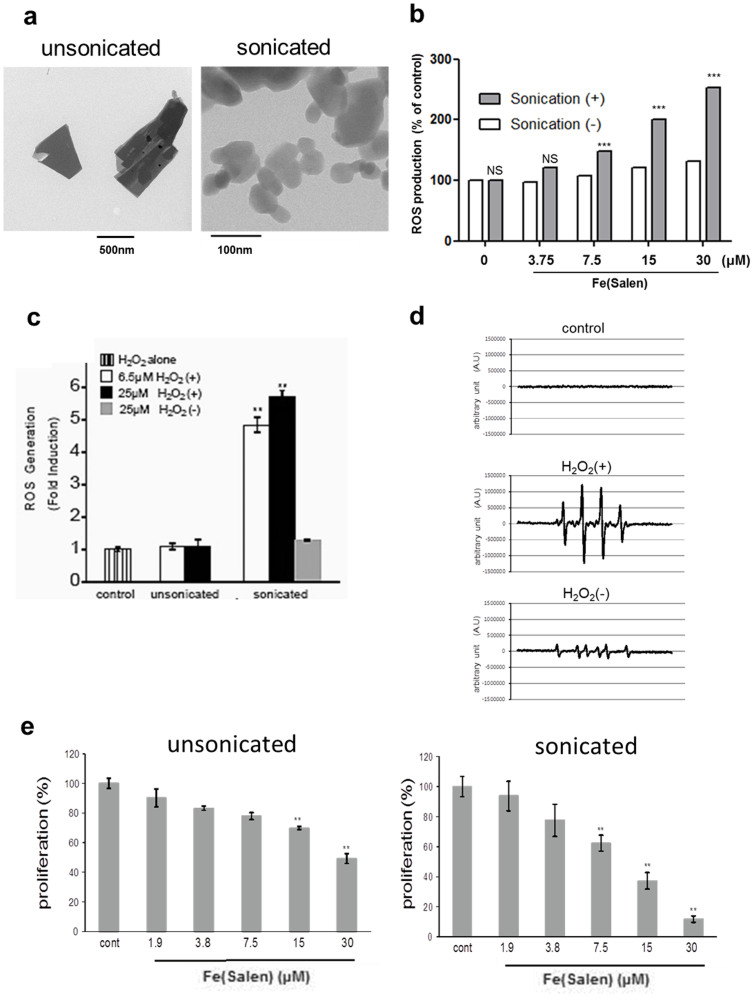
Mechanism underlying the anti-cancer effect of Fe(Salen). (a), TEM images of initial (*unsonicated*) and extensively sonicated (6 hours) Fe(Salen) particles (*sonicated*). Extensive sonication at 4°C for 6 hours (Sonifier 250, Branson, 2 kHz) broke up Fe(Salen) particles, as determined by TEM. A scale bar is shown at the bottom. Note that the particle size was reduced, with decreased density, and that the edges of the particles were smoothed compared with those of the unsonicated particles. Unsonicated sample presents sharp and irregular particulate with size of a few hundred nanometers to few micrometers while sonicated particles shows more smoothened and thin morphology. Most likely, sonication destroyed nanoparticles, leading to the reduction of the particles size (see Fig. S4) and their density, and their edges were smoothened. X-ray structural analyses on BL32XU at SPring-8 have revealed that these nanoparticles have a crystalline structure (Fig. 5). (b), Effect of sonication on ROS generation in cell culture. ROS generation was measured in the presence of sonicated Fe(Salen) particles using the fluorescent dye 2',7'-dichlorofluorescein diacetate (DCFH-DA) in MAT-Lu prostate cancer cells (n = 6, NS, not significant, ***p < 0.001 vs. control). (c), Effect of sonication on ROS generation *in vitro*. ROS generation was compared between unsonicated and sonicated Fe(Salen) particles (6.5 or 25 μM) using the fluorescent dye 2',7'-dichlorofluorescein diacetate (DCFH-DA) in the absence of cells. ROS generation was measured immediately after adding hydrogen peroxide. ROS generation was small with unsonicated Fe(Salen), but substantial with sonicated Fe(Salen); however, this difference was only observed in the presence of hydrogen peroxide. (n = 4, **p < 0.01 vs. control). (d), ESR analysis of ROS production. ROS production was evaluated using an EMX-8/2.7 ESR spectrometer (Bruker Biospin, Billerica, MA, USA) with sonicated Fe(Salen). *Control*; 10 μM hydrogen peroxide alone, *H_2_O_2_(+)*;Fe(Salen) in the presence of hydrogen peroxide, *H_2_O_2_(−)*;Fe(Salen) in the absence of hydrogen peroxide. Note that hydrogen peroxide was required to produce ROS. (e), Cytotoxicity. MAT-Lu were incubated with unsonicated or sonicated Fe(Salen) at 37°C for 24 hours. Cell viability was determined by means of XTT assay. (n = 4, *p < 0.05, **p < 0.01 vs. control). Note that cytotoxicity was greater with sonicated Fe(Salen) (*lower*).

Because this iron-salen is difficult to solubilize, we used it as particles suspended in water or saline after sonication. Transmission electron microscopy (TEM) showed that sonication for 6 hours reduced particle size, with decreased density, and that the edges of the particles were smoothed compared with those of the unsonicated particles (Fig. 2a). The size distribution of the colloidal suspension of Fe(Salen) particles was evaluated using dynamic light scattering measurements (Fig. S4a and b). The unsonicated and sonicated Fe(Salen) particles show size distributions from 1.2–3 μm and 60–800 nm, respectively, in agreement with the TEM results. The stability of the colloidal suspension of sonicated Fe(Salen) particles was also determined by zeta potential measurements. Fe(Salen) particles show a zeta potential value of −24.1 mV, indicating a stable colloidal dispersion^16^.

### Mechanism of cytotoxicity

We first examined whether Fe(Salen) particles were indeed taken up by culture cells. We used transmission electron microscopy (TEM), as well as energy-dispersive X ray spectroscopy (EDS), to determine such uptakes^17^,^18^. TEM revealed that Fe(Salen) particles were found within VX2 cells, and EDS demonstrated that such particles were indeed Fe(Salen) (Fig. S8).

The molecular mechanisms of other metal-salens, such as chloro(*N*,*N*′-ethylenebis(salicylideneaminato))iron ([Fe(salen)]Cl), have been suggested to involve production of reactive oxygen species (ROS)^11^,^12^. In agreement with those reports, we found that the *N*,*N*′-bis(salicylidene)ethylenediamine iron particles after sonication induced ROS production in MAT-Lu cultured cells (Fig. 2b). Sonication of Fe(Salen) induced DNA nicking using super coiled plasmid DNA experiment (Fig. S7a (*left*)). Such DNA nicking was similar to that of cisplatin^19^ (Fig. S7a (*right*)). Sonication of Fe(Salen) also increased caspase 3/7 in MAT-Lu cells (Fig. S7b). Fe(Salen) -induced cytotoxicity was negated by Vitamin C which was well known to antioxidant^20^,^21^ in MAT-Lu cells (*data not shown*). Moreover, ROS production was increased even in the absence of cells when the native particles were sonicated and H_2_O_2_ was added immediately (Fig. 2b, c and d).

It is widely accepted that iron ion, either divalent or trivalent, can react with hydrogen peroxide to produce ROS via the Fenton or Fenton-like reaction, respectively^22^,^23^. Because the *N*,*N*′-bis(salicylidene)ethylenediamine iron particles contains iron, this may be the mechanism of ROS production and, potentially, cytotoxicity. Sonicated the *N*,*N*′-bis(salicylidene)ethylenediamine iron fine particles showed greater cytotoxicity than unsonicated particles (Fig. 2e), suggesting that the production of ROS becomes greater when particle sizes become smaller by sonication. This may be reasonable because iron salen molecules can be eluted more when large particles are broken into smaller one. It is well known that cancer cells produce larger amounts of hydrogen peroxide than normal cells^24^ in cellular mitochondria and peroxisomes^25^, so the effect of this salen might be greater on cancer cells than normal cells. To compare between the production of cancer cells and normal cells, we measured ROS production in the presence of Fe(Salen) in MAT-Lu cells and rat aorta smooth muscle cells. This result demonstrated that ROS generation effect of Fe(salen) might be greater on cancer cells than normal cells. Putting together, ROS may play an important role, at least, in inducing cytotoxicity of Fe(Salen) although we do not deny the presence of additional mechanisms for cytotoxicity.

### Magnetic property

**Figure 3:**
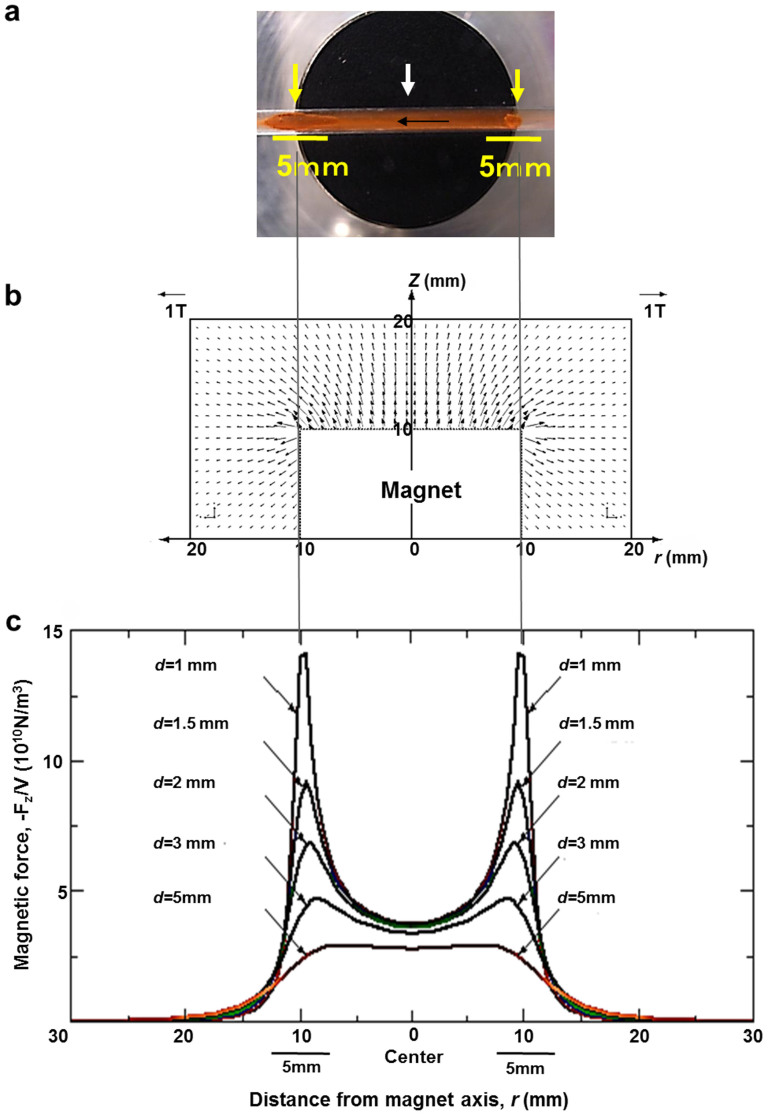
Magnetic entrapment of Fe(Salen) particles in flowing water. (a), Trapped Fe(Salen) particles in flowing water (also see Movie S1). A permanent bar magnet was placed under a glass tube to trap Fe(Salen) particles in flowing water. Note that Fe(Salen) accumulated more at the edges (*yellow arrows*) than at the center (*white arrow*) of the magnet. Accumulation on the left edge was greater than that on the right because the water flow was from right to left. (b), Calculated magnetic field (magnetic flux density) distribution around the top of the magnet. *r* and *z* denote the distance from the magnet axis and the distance from the top of the magnet, respectively. (c), Calculated magnetic force for unit volume of Fe(Salen) or its cluster, for which a spherical shape was assumed. The magnetic force component parallel to the magnet axis (*F*_z_) divided by the volume of the particle (*V*) is plotted against the distance from the magnet axis (*r*). *d* is the distance from the top surface of the magnet. Note that the magnetic force was greatest at the magnet edges (5 mm), where Fe(Salen) was indeed trapped in the flowing water (a).

Our preliminary *in silico* analysis using the first principles calculations^15^ implicated the possible presence of magnetisms in *N*,*N*′-bis(salicylidene)ethylenediamine iron (see Methods). We then found that the *N*,*N*′-bis(salicylidene)ethylenediamine iron particles were indeed attached to a bar magnet in air. To examine this attraction more precisely, we analyzed the movement of crystalline particles of the *N*,*N*′-bis(salicylidene)ethylenediamine iron particles under a microscope. As shown in the movies (Movies S1a, b, and c), similarly sized particles with different shapes were all attracted to the magnet with similar velocity under a microscope. Thus, the magnetic property, at the least, does not depend upon the shape of crystalline particles. We also examined the attachment to a bar magnet in a simulated blood (water) flow (Fig. 3a and Movies S2). The particles were accumulated at the edges of the permanent magnet, where the calculated magnetic field was greatest (Fig. 3a, b and c).

**Figure 4:**
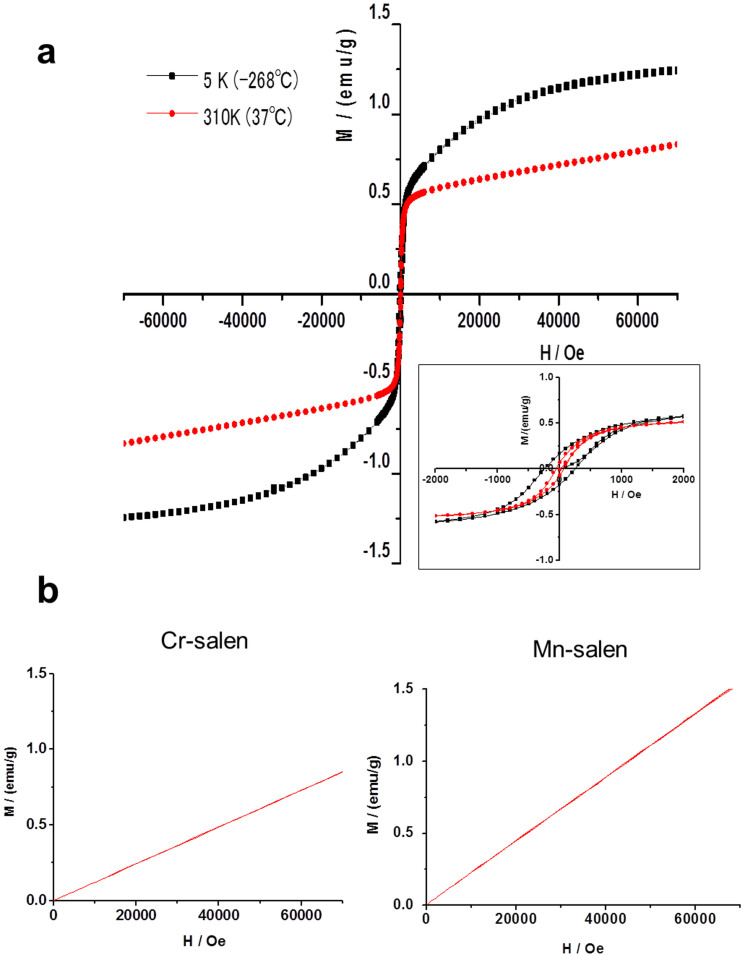
Plots of magnetization versus magnetic field for Fe(Salen) and other salen derivatives. Magnetization versus magnetic field curve values was generated using a superconducting quantum interference device (SQUID) (Quantum Design MPMS7 system). Each derivative was enclosed in a plastic capsule (Quantum Design) for measurement. The magnetization versus magnetic field curve of Fe(Salen) was plotted from −268°C (5 K) to 37°C (310 K) (a). Changes in magnetization (M/(emu/g)) with applied magnetic field (H/Oe) between −70,000 and 70,000 Oe are shown. Fe(Salen) particles were stable and retained the same magnetization versus magnetic field curve at 37°C for at least three years in air. *Inset*: Magnified view of changes in magnetization (M/(emu/g)) with applied magnetic field (H/Oe) between −2,000 and 2,000 Oe. (b, c) Changes in magnetization with applied magnetic field (H/Oe) between 0 and 70,000 Oe are similarly plotted against magnetic field for Cr(II)-salen and Mn(II)-salen at 37°C (310 K).

In order to further characterize the magnetic properties, we measured the magnetization versus magnetic field curves using a superconducting quantum interference device (SQUID) (Quantum Design MPMS7) system, i.e., the standard method to identify magnetism. Magnetization versus magnetic field plots from −268°C (5 K) to 37°C (310 K) revealed that the compound exhibited magnetically active (Fig. 4a) with hysteresis loop (Fig. 4a *insert*). The magnetization of this iron-salen was found to be stable in air as well as in solution. The magnetic properties remained unchanged for at least three years. These data, coupled with the results of elemental analysis and mass spectroscopy (Supplementary Table 1), strongly suggest that this iron-salen is a magnetic compound. For comparison, Cr- and Mn-salen also exhibited positive magnetization with increasing applied magnetic field. However, they showed weaker magnetization at 37°C (310 K) (Fig. 4b and c). In contrast, another iron-salen, i.e., [Fe(salen)]Cl was cytotoxic^13^,^14^, but does not have enough magnetization value for drug delivery in response to magnetic field (*data not shown*). Accordingly, we considered that our iron-salen might act as an intrinsic magnet and have potential for drug development.

To exclude the presence of unexpected magnetic metals in the *N*,*N*′-bis(salicylidene)ethylenediamine iron particles, we performed impurity analysis. *N*,*N*′-bis(salicylidene)ethylenediamine iron particles was commercially purchased from a chemical company (Tokyo Chemical Industry Co., Ltd, Tokyo). However, we did not detect the presence of any magnetically relevant metals, except for iron, in the *N*,*N*′-bis(salicylidene)ethylenediamine iron particles (Supplementary Table 1). We also newly synthesized *N*,*N*′-bis(salicylidene)ethylenediamine iron particles^26^,^27^. Synthesized salen iron was similarly attracted to a magnet and exhibited anti-cancer property, as commercial one.

### Crystallographic analysis

**Figure 5:**
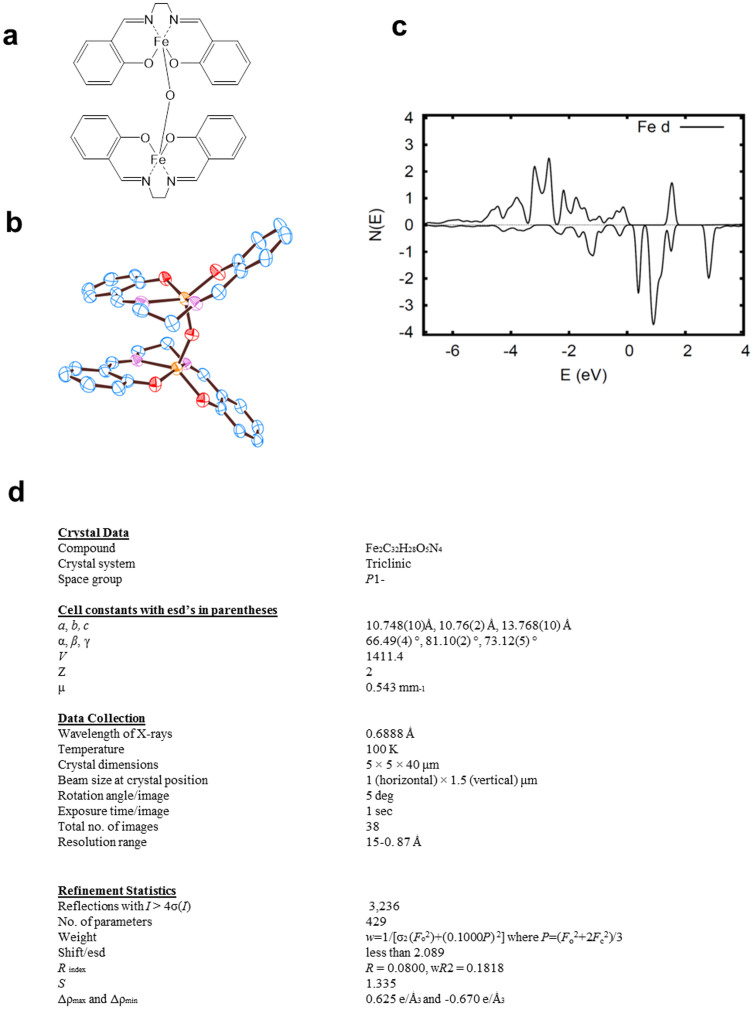
Structure of Fe(Salen). (a). Chemical structure of Fe(Salen). (b). ORTEP. The crystal structure of Fe(Salen). Atomic anisotropic B-factors for non-hydrogen atoms are shown as ellipsoids. Blue, red, purple, and orange atoms represent carbon, oxygen, nitrogen, and iron, respectively. Hydrogen atoms were omitted for clarity. See also Supplementary Movie S3. (c). Projections of the spin-polarized density of states onto Fe *d* orbitals within the LAPW sphere radius of 1.6 bohr. The results of PBE GGA calculations with relaxed atomic positions on a per-atom basis are shown. The majority spin is shown above the axis, and the minority is shown below. Note the broader majority spin features reflecting strong spin-dependent hybridization. (d). Crystallographic data and statistics. For details of data processing and refinement, see METHODS.

It is highly unusual for an organic-based molecular compound to be magnetic above room temperature. To understand the structural basis of this finding, we performed single-crystal X-ray diffraction analysis of iron-salen (Fig. 5a, b and d). The crystallographic data indicated that two iron-salen units are bonded at Fe by a single oxygen atom (Fig. 5a and b). Thus, this *N*,*N*′-bis(salicylidene)ethylenediamine iron particles is in fact μ-oxo *N*,*N*′-bis(salicylidene)ethylenediamine iron or [Fe(salen)]_2_O (Fe(Salen)) on a strict definition. It is most likely that *N*,*N*′-bis(salicylidene)ethylenediamine iron was quickly oxidized in air to give Fe(Salen)^28^,^29^.

Importantly, we found that the Fe-O-Fe angle was 146.359° (Movie S3). This angle is theoretically consistent with a ferromagnetic interaction, as known for other ferromagnetic crystals, such as YTiO_3_^30^. It agrees with the classic Goodenough-Kanamori-Anderson rule^31^ that determines magnetic interactions of a transition metal ion, such as Fe or Ti, through O. It is most likely that this Fe-O-Fe angle structure plays an important role in generating magnetism in Fe(Salen). Of note, this angle was maintained over the temperature range of −160°C and 37°C; this finding is consistent with the SQUID observation of magnetism over a wide temperature range (−268 and 37°C) (Fig. 4a).

### First principles calculations

We further analyzed the presence of magnetisms by first principles calculations with the findings from X-ray crystallographic study (Fig. 5c and Fig. S1). In this case, we used the GGA+U method^32^ and the CASTEP program package^33^ using the experimental crystal structure obtained from the above experiment. We calculated the energy difference between the ferromagnetic ordering and two different antiferromagnetic orderings of the Fe spins in the four Fe atom unit cell. We found that ferromagnetism was indeed preferred based on the calculated energies. Further, we have found that the magnetic moments is distributed over the salen units by the Fe-induced spin-dependent, sequential hybridization of N, O and C, and that the magnetic moment is not simply localized at the Fe ions (for detailed results, see Supplementary File 1).

Putting together, the above characterizations, i.e., magnetic attraction, SQUID analysis, X-ray crystallographic findings, and first principles calculations, have strongly suggested the presence of magnetism with this iron-salen (Fe(Salen)).

### Magnet-guided drug delivery in cultured cells

If Fe(Salen) magnetic and cytotoxic, the cytotoxic property should be delivered, at the least, by magnetic attraction. We thus examined magnet-guided delivery of the Fe(Salen) particles using a permanent magnet. According to the principle of magnetic physics, large particles are efficiently attracted by magnet^34^. However, to improve ROS production and thus anti-cancer activity, sonicated particles may be preferred (Fig. 2). Accordingly, we used Fe(Salen) particles that had been sonicated for 30 min, empirically, throughout our experiments.

**Figure 6:**
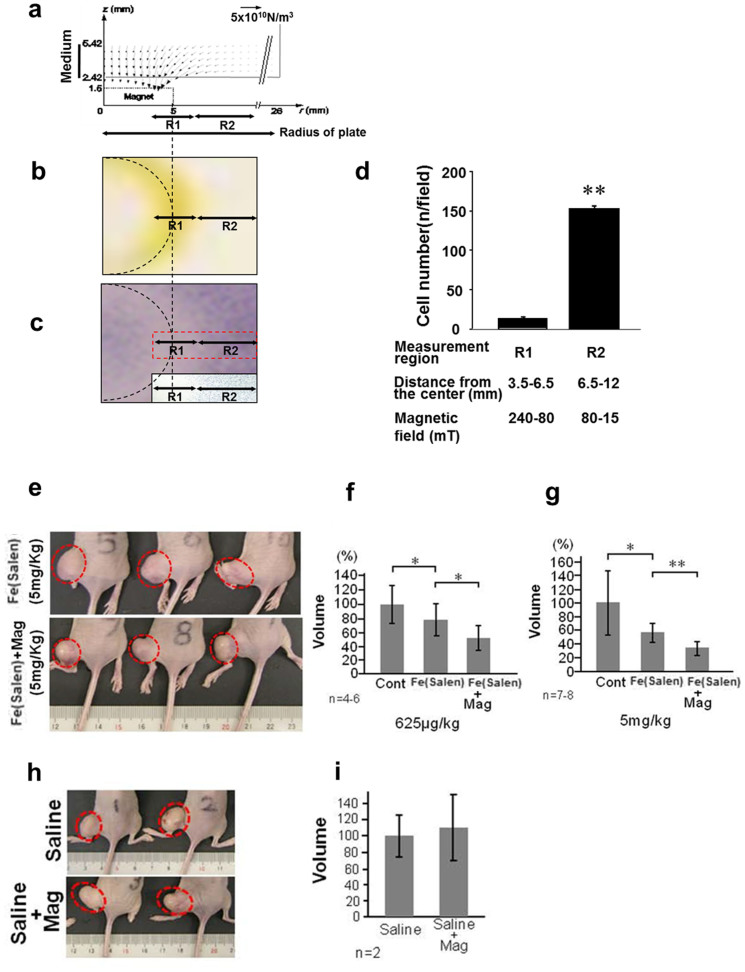
Magnet-guided drug delivery in cultured cells and mice. (a–d), Drug delivery in cultured cells. Clone M3 were cultured in the presence of Fe(Salen) (50 μM), and a flat, round magnet was placed beneath the center of the dish for 24 hours. The calculated magnetic force per unit volume of Fe(Salen) or its cluster (assuming a spherical shape) at various distances from the top surface of the magnet was plotted against the distance from the axis of the magnet (a). *r* and *z* denote the distance from the magnet axis and the distance from the top of the magnet, respectively. Based on the estimated magnetization of Fe(Salen) particles (0.5 emu/g at 37°C), the magnetic force (*F*_M_) attracting Fe(Salen) was calculated according to the following equation: *F*_M_ = *VM*_s_ *gradH*, where*V* (m^3^), *M*_s_ (T) and *H* (A/m) are the volume of the particle, saturated magnetization of the particle, and applied magnetic field, respectively. The accumulation of the Fe(Salen) fine particles (b) and stained cells (c) is shown (*broken semicircle* shows the edge of the magnet). The number of cells was determined at the edge (R1) or at various distances (R2) from the magnet (n = 10, **p < 0.01) (d). (e–i), Drug delivery in mice. Leg tumors were established by grafting MAT-Lu cells into mice. A bar magnet (630 mT, surface magnetic flux density) was used for delivery. Effect of Fe(Salen) with magnet application (e). Fe(Salen) (5 mg/kg) (*Fe(Salen)*) was intravenously injected every day in the presence or absence of magnet application for 14 days. Note that the tumor size was decreased when the magnet was applied (*bottom*) compared with when it was not (*upper*), at the same dose of Fe(Salen). Dose-dependent effect of Fe(Salen), 625 μg/kg (f) and 5 mg/kg (g). *Control*, only saline; *Fe(Salen)*, Fe(Salen) without magnet application; *Fe(Salen)+Mag*, Fe(Salen) was administered, and a magnet was applied. (n = 4–6 (625 μg/kg) (f), or n = 7–8 (5 mg/kg) (g), *p < 0.05, **p < 0.01). Effect of magnet application (h and i). A magnet was applied for 3 hours every day in the absence of Fe(Salen) for 14 days. Note that magnet application did not affect the tumor growth. (n = 2).

Fe(Salen) particles were added to melanoma cells in culture (Fig. 6a–d). A flat, round magnet (240 mT) was then placed beneath the culture dish for 24 hours. The melanoma cell death was greatest at the edge of the magnet, where the magnetic field was maximal, and smallest at locations furthest from the magnet (Fig. 6a). This pattern of activity corresponded well to the accumulation pattern of Fe(Salen) *per se* (Fig. 6b). Thus, the strength of the magnetic field correlated with the delivery of its anti-cancer effect (Fig. 6c and d). Cell numbers were similar throughout the dish when the magnet was applied in the absence of Fe(Salen) or when Fe(Salen) was applied in the absence of the magnet (*data not shown*).

### Magnet-guided drug delivery in mouse

To examine the anti-cancer effects of Fe(Salen) and the feasibility of magnet-guided delivery in intact animals, we used a mouse model, in which prostate cancer cells (MAT-Lu) were grafted onto the legs (Fig. 6e–i). Fe(Salen) particles were sonicated, suspended in saline, and injected via the tail vein. A bar magnet (630 mT) was then placed on top of the leg tumor for three hours every day. After 14 days, we found that the intravenous injection of Fe(Salen) alone decreased tumor size, confirming its anti-cancer effect *per se*. This anti-cancer effect was modest, but significant and dose-dependent (625 μg versus 5 mg/kg/day). More important, it was augmented modestly, but significantly, by magnet application to the leg tumor (Fig. 6f and g). Application of the magnet in the absence of Fe(Salen) had no effect (Fig. 6h and i).

We then used Fe(Salen) to treat melanoma tumors grafted on the tails of the mice. Although melanoma was not highly Fe(Salen) -sensitive (Fig. 1g), we used the melanoma model because it was very easy to evaluate the spread of pigmentation on the tail. We also improved the method of magnet application.

**Figure 7:**
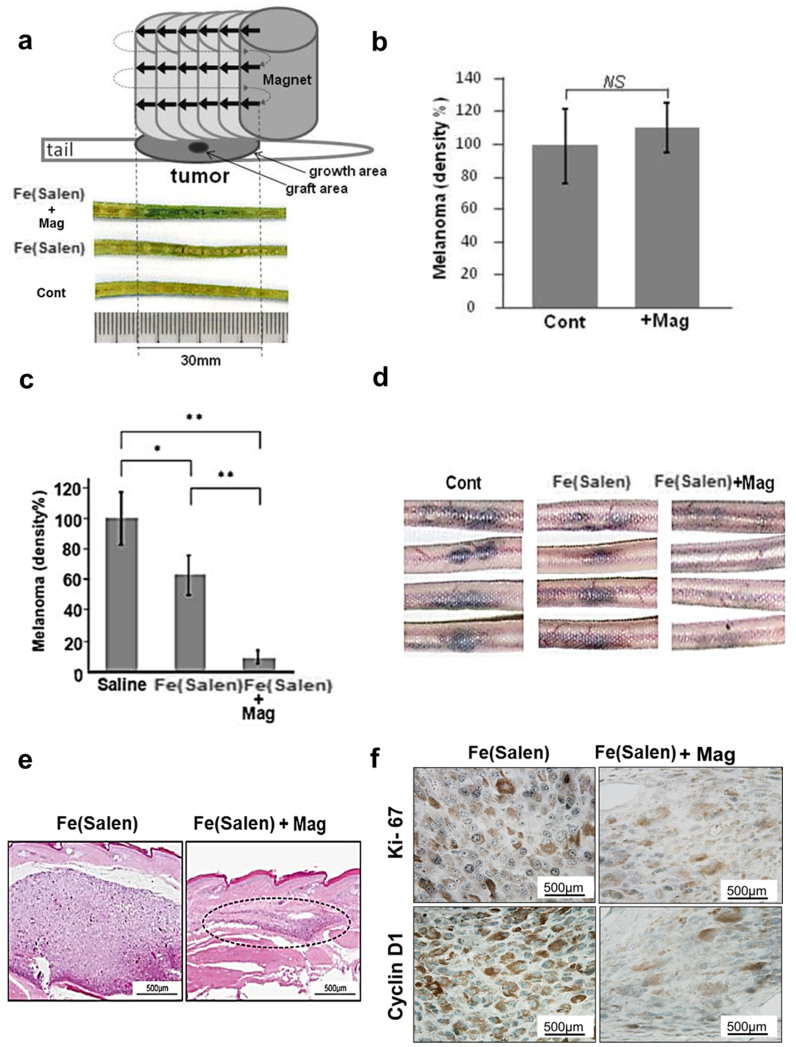
Magnet-guided delivery by the sliding method with a stationary magnet. (a–b), The scheme illustrates the sliding method with a stationary magnet (a). The magnet was applied to the edge of the expected melanoma growth area for 10 minutes. The magnet was slid by 5 mm along the tail (arrows) and similarly applied for 10 minutes (*upper*). This magnet sliding was repeated 5 times over the length of 30 mm for 3 hours after each injection. Note that the magnet was moved by 5 mm at each sliding because the magnetic field generated by this magnet was strongest within 5 mm of its edge (see Fig. 1). Longitudinal sections of mouse tails were chemically stained for Fe(Salen) by Prussian blue (*lower*). Note that the area of Fe(Salen) accumulation is densely stained in blue (30 mm in length), following the magnet-guided drug delivery. *Fe(Salen)+Mag*, Fe(Salen) administration (50 mg/kg/day) with magnet application; *Fe(Salen)*, Fe(Salen) administration without magnet application; *Cont*, saline alone. Effect of magnet sliding by itself (b). The sliding of the magnet in the absence of Fe(Salen) (*+Mag*) did not affect melanoma growth. (n = 4). (c–f), Effect of magnet-guided drug delivery with the sliding method on mouse tail melanoma model. Melanoma was established in mouse tail. Fe(Salen) (50 mg/kg) was administered intravenously, and a magnet (630 mT) was applied to the melanoma site (also see Fig. 5a and b). This process was repeated for 14 days, and the extension of melanoma pigmentation was evaluated. Comparison of the area of melanoma extension (n = 8, *p < 0.05, **p < 0.01 vs. control) (c). Representative photos of mouse tails with melanoma pigmentation (d). Note that mice in the *Fe(Salen)* and *Fe(Salen)+Mag* groups received the same amount of Fe(Salen). Histochemical analysis of melanoma with HE staining (e). The broken circle shows the putative area of melanoma. Tissue sections were immunostained with anti-Ki-67 antibody (*Ki-67*) and anti-cyclin D1 antibody (*Cyclin D1*) (f). Representative photos are shown. Melanin pigments are shown in brown, and immunopositive signals are shown in blue-black. Note that intensity of both stains was decreased in the *Fe(Salen)+Mag* group. *Fe(Salen)*, Fe(Salen) alone; *Fe(Salen)+Mag*, Fe(Salen) with magnet application.

Fe(Salen)was injected into the *proximal* tail vein of the mice, and a magnetic field was applied to the *distal* tail lesion where the melanoma was grafted; this was to avoid the immediate trapping of Fe(Salen) by magnet after injection. We then modified the method of magnet application in this model, after many tries and errors, so that Fe(Salen) could be accumulated over a greater area, not just at the point of magnet application. Specifically, the magnet was first applied to the edge of the expected tumor growth area on the tail, and then swept multiple times, so that the magnetic field was spread over a greater area of the tail. Efficient accumulation of Fe(Salen) was confirmed by chemical staining of Fe(Salen) in tail tissues (Fig. 7a). Magnet application by itself did not alter tumor growth (Fig. 7b).

This process was repeated for 14 days, and the extension of melanoma pigmentation was evaluated using NIH J imaging software^35^,^36^. The melanoma extension was worst in the control group (100 ± 17.2%), which received only saline. The extension was modestly, but significantly decreased (63.7 ± 16.3%) in the group that received Fe(Salen), confirming its anti-cancer effect *in vivo*. The melanoma largely disappeared (9.1 ± 3.4%) in mice that received both Fe(Salen) and magnet application (n = 6–10 per group) (Fig. 7c and d). Importantly, the latter two groups received the same dose of Fe(Salen), however, there was a marked difference in tumor growth, reflecting the magnet application *per se*. This was also confirmed in HE staining (Fig. 7e) as well as by immunohistochemical staining of Ki-67 and cyclin D1, markers of melanoma proliferation^37^ (Fig. 7f).

### Visualization of drug accumulation using MR imaging

**Figure 8:**
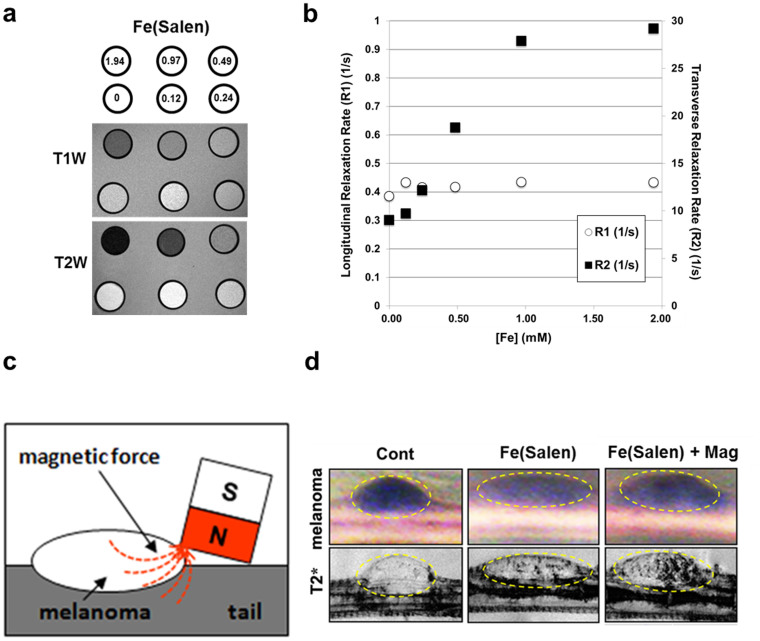
MR imaging of Fe(Salen) *in vitro* and *in vivo* studies. (a–b), MR imaging by sample study. A sample of each concentration of Fe(Salen) particles (0–1.94 mM) was acquired with a 7-Tesla scanner for T1- and T2-weighted images (a). Proton longitudinal (R1) and transverse relaxation rate (R2) of Fe(Salen) were calculated quantitatively (b). (c–d), MR imaging *in vivo* study. A schematic figure of magnet application to half of the melanoma tumor is presented (c). A permanent magnet (630 mT, surface magnetic flux density) was placed in contact with half of the tumor for 3 hours, and MR imaging was performed. Sagittal slice of T2*-weighted MR images at the tail were obtained (T2*) (d). At the Fe(Salen) administration with magnet application (*Fe(Salen)+Mag*), signal reduction at half side of the tumor on T2*-weighted MRI was observed. Representative photos are shown.

Because of its magnetism, Fe(Salen) particles can be visualized by MR imaging. *In vitro* sample of the Fe(Salen) particles(Fig. 8a) exhibited concentration-dependent (0–1.94 mM) negative signal alteration on a T2-weighted image, indicating that this anti-cancer compound has capability for MRI visualization. Although slight signal enhancement in the T1-weighted image was observed at 0.12 mM (Fig. 8a), the signal alterations were also negative depending on the concentration, presumably due to the T2 or T2* shortening. It also demonstrated that slight longitudinal relaxation rate increment (R1 = 0.16 ± 0.16 s−1 mM^−1^) and moderate transverse relaxation rate enhancement (R2 = 14.49 ± 6.74 s−1 mM^−1^) determined quantitatively (Fig. 8b, 0.12–0.97 mM concentration range was used for the calculation due to the linearity). To examine MR imaging of the Fe(Salen) particles after magnet-guided drug delivery in mouse, the Fe(Salen) particles were intravenously administered to melanoma-grafted model on the tails and put a stationary magnet on half side of the tumor (Fig. 8c). The tails were immediately removed and set to a 9.4 tesla MR imaging system (vertical bore magnet). The stationary magnet induced signal reduction at half side of the tumor on T2*-weighted MRI indicating local accumulation of Fe(Salen) (Fig. 8d).

## Discussion

Cytotoxic effect of various salen derivatives have been demonstrated in recent studies^11^,^12^,^13^,^14^. In this study, we found that Fe(Salen), which has not been examined in previous studies, was similarly cytotoxic. Unexpectedly, this salen derivative was readily attracted by a stationary magnet, and showed magnetization with a superconducting quantum interference device. Fe(Salen) was obtained as *N*,*N*′-bis(salicylidene)ethylenediamine iron (II), but, most likely, was quickly oxidized to μ-oxo *N*,*N*′- bis(salicylidene)ethylenediamine iron ([Fe(salen)]_2_O) as shown by X-ray crystallography. This led to the formation of the Fe-O-Fe angle of 146.359° (Movie S3), and generated, at least in part, magnetism in Fe(Salen). The first principles calculations also exhibited that the magnetic moments is distributed over the salen units and that the magnetic moment is not simply localized at the Fe ions. We do not know whether additional mechanisms may exist to generate magnetism in this compound.

Nevertheless, we have found that Fe(Salen) particles are cytotoxic, and that cytotoxic property can be delivered, by a magnet, to a desired location, as an anti-cancer drug, in cultured cells and in various animal cancer models. Thus, the first advantage of this strategy is that such a drug can be delivered to the tumor in a magnet-guided manner, and may cause less adverse effects on non-targeted healthy tissues. Our preliminary study has demonstrated that accumulation increased by more than 10-fold by a stationary magnet (*unpublished data*). It is thus considered that the presence of magnetic property in Fe(Salen) significantly increases the efficacy of anti-tumor property as indicated in our study. Because magnetic and anti-cancer properties were contained in a single drug molecule in the case of Fe(Salen), this is distinct from the conventional drug-laden magnetic method, in which drug molecules and magnetic molecules are packaged in micelle. Fe(Salen) is also resistant to high temperature (*unpublished data*), unlike micelle or liposome particles.

Secondly, Fe(Salen) particles *per se* were visualized by MRI, and its accumulation was demonstrated by MRI after drug delivery in the cancer animal model. It has been difficult to determine the appropriate drug doses for cancer chemotherapy^38^, as well as to assure its delivery to the tumor. Drug doses have been determined empirically, using the body surface area index of each patient, at least in the past half-century^39^,^40^. It has been used regardless of age, sex, or organ function of each patient^38^. Although this index does not assure delivery and thus proper doses of the drug to the tumor, it is commonly used in many clinical practices due to a lack of superior alternatives. Accordingly, cancer chemotherapy may fail, occasionally, from either drug over- or under-dosing. Magnetic anti-cancer drugs, as demonstrated in our study, can be visualized as MR imaging contrast; therefore, it may be possible to assure drug delivery to the tumor and to even quantify the amount of drug in cancerous and normal tissues in future. Accordingly, such drug molecules with magnetic property might enable us to develop powerful anti-cancer therapeutic and imaging strategies^41^,^42^. Furthermore, our findings might be utilized to magnetize other anti-cancer drugs. Covalent linkage of the magnetic core of Fe(Salen) to an existing anti-cancer drug, such as paclitaxel, may be designed, leading to the generation of a new compound, which possesses the magnetic property in addition to the classic cytotoxic property of paclitaxel (Umemura *et al.* *unpublished observation*).

The first principles calculations have been used as nano-material simulation in various industrial fields, such as aerospace industry^15^; this is to search for a metal material(s) from numerous candidate materials, so that the final material exhibits specific physical function for a space craft^43^. This process is similar to a drug screening procedure. It searches for a drug compound(s) from a large compound library through virtual-docking computer simulation, so that the final compound may have desired pharmacological function for medical therapy. In this regard, we have applied a method in aerospace industry to drug screening, leading to identifying a magnetic anti-cancer compound. We believe that such a cross-industrial use of screening technique may be useful to find a drug with novel property in pharmacological research.

Putting together, Fe(Salen) may be useful for enabling magnet-guided delivery of itself and, potentially, many other anti-cancer agents. It would thus provide an effective tool to improve treatment efficacy and to decrease the required dose for chemotherapy. Such anti-cancer therapeutic and imaging strategies^42^ might alter our concept of future pharmacotherapy and anti-cancer drug development.

## Methods

### Reagents

Salen was purchased from Sigma-Aldrich (St. Louis, MO, USA). The commercially available A *N*,*N*′-bis(salicylidene)ethylenediamine iron particles (TCI product code D2571) was purchased from Tokyo Chemical Industry Co., Ltd. (Tokyo, Japan) and used as received or after sonication. We also synthesized this iron salen and other salen derivatives, such as [Fe(salen)]Cl, Cr(salen) and Mn(salen)^26^,^27^. Purchased and synthesized iron-salen showed the same physical and biological properties. *N*,*N*′-bis(salicylidene)ethylenediamine iron(II) (CAS# 14167-12-5) was most likely quickly oxidized to give [Fe(salen)]_2_O (Fe(Salen)) (CAS# 18601-34-8) as shown by X-ray crystallographic analysis. Salen compounds were used as suspensions, after extensive sonication for six hours, in normal saline for animal studies and in very low concentrations of Dimethyl sulfoxide (DMSO) (0.02%) for cellular assays because of poor solubility.

### Magnet

A flat, round magnet (240 mT surface magnetic flux density, neodymium-based magnet of grade N50, 10 mm in diameter) was purchased from Shin-Etsu Chemical Co., Ltd. (Tokyo, Japan) in Fig. 6.

### X-Ray crystallographic analysis

A dark brown prismatic crystal of iron-salen with approximate dimensions of 0.005 × 0.005 × 0.03 mm was sealed in a thin-walled quartz capillary. Diffraction data were collected at 100 K on BL32XU at SPring-8 (RIKEN, Hyogo, Japan) using the helical data collection method^44^, with a Rayonix MX225HE X-ray CCD detector. In this method, the irradiation position of X-rays on the crystal is gradually translated during data collection. The diffraction data were processed with the *HKL2000* package and scaled with the program *SCALEPACK*^45^.

The structure was then solved by an iterative dual-space direct method and refined anisotropically using the *SHELX* package^46^. Hydrogen atoms were located by difference Fourier synthesis and included in the subsequent refinement with isotropic temperature factors. Hydrogen atoms that were not observed in the difference Fourier map were located at calculated positions. The final *R*-factor of the structure converged to 0.063 for 3,236 reflections with *I* > 4σ(*I*). The crystallographic data (excluding structure factors) for the structure reported in this paper have been deposited with the Cambridge Crystallographic Data Centre as supplementary publication no. CCDC- 906837.

### First principles calculations

In preliminary analysis, we first identified the highest charge spin density of various compounds containing a magnetite (Fe_3_O_4_) cluster that consists of 103 atoms (Fe_39_O_64_). The magnetite cluster was modeled after the crystal structure of Fe_3_O_4_ magnetite (*Fd3m*, No. 227 in the International Tables, a_0_ = 0.839 nm). We then looked for those compounds that satisfy a condition for potential pharmacological development^47^.

For detailed analysis, the magnetism and electronic structure of iron-salen were studied by first principles calculations with generalized gradient approximation plus Hubbard U (GGA+U)^32^, followed by electronic structure optimization using Ensemble Density Functional Theory (EDFT)^48^. These calculations were done using the CASTEP program package^33^. GGA calculations of the spin density, electronic structure and related properties were performed using PBE-GGA as implemented in the WIEN2k code. The WIEN2k calculations were done with optimization of internal atomic coordinates in the unit cell based on total energy minimization.

### Cell lines and cell culture

Mouse melanoma clone M3 cells (Cloudman S91 melanoma, American Type Culture Collection (ATCC)) were cultured in F-10 medium supplemented with 15% normal horse serum (NHS) and 2.5% fetal bovine serum (FBS). Rat prostatic adenocarcinoma cells (R3327-MAT-Lu, kindly provided by Dr. J. T. Isaacs, Johns Hopkins University, MD) were cultured in RPMI-1640 medium supplemented with 10% FBS and 250 nM dexamethasone. Rabbit squamous cell carcinoma (VX2) and mouse osteosarcoma (POS-1) cells (kindly provided by the Cell Resource Center for Biomedical Research, Tohoku University, Japan and by Dr. Y. Nakamura, Kanagawa Cancer Center Research Institute, Japan, respectively) were cultured in RPMI-1640 medium supplemented with 10% FBS.

### Sodium 2,3,-bis(2-methoxy-4-nitro-5-sulfophenyl)-5-[(phenylamino)-carbonyl]-2H-tetrazolium inner salt (XTT) assay

Cell proliferation assay was performed using a commercially available kit, XTT Cell Proliferation Assay Kit (ATCC, Virginia, U.S.A.) according to the manufacturer's protocol^49^.

### Measurement of reactive oxygen species (ROS)

Measurement of ROS was performed as we previously reported^20^. MAT-Lu cells were plated in 96-well culture plates overnight. Cells were then treated with 0, 3.6, 7.5, 15, and 30 μM Fe(Salen) before/after sonication for 6 hours at 37°C for 24 hours. The intracellular ROS level was then measured using a fluorescent dye, 2′,7′-dichlorofluorescein diacetate (DCFH-DA; Sigma, Japan). ROS production was measured using a microplate reader equipped with a spectrofluorometer (ARVO-Mx, PerkinElmer, Massachusetts, U.S.A.) at an emission wavelength of 538 nm and extinction wavelength of 485 nm.

### Apoptosis assays

TdT-mediated dUTP nick-end labeling (TUNEL) assay was performed using Terminal Deoxynucleotidyl Transferase-Mediated dUTP Nick-End Labeling (Promega, WI, USA) according to the manufacturer's protocols^49^.

### Magnet-guided delivery of Fe(Salen) particles in mice

#### Leg tumor model

MAT-Lu cells (prostate cancer cells, 5 × 10^6^ cells) were grafted into the legs of BALB/c Slc-*nu/nu* mice by subcutaneous injection (6–7 weeks old; 6–10 mice/group) (SLC, Shizuoka, Japan) as previously shown^50^. Preliminary studies showed that MAT-Lu cells grew to form bumpy masses in the legs. Three days after grafting, intravenous injection of Fe(Salen) particles were initiated into the tail veins of the mice as a daily single dose (625 μg/kg, n = 4–6 or 5 mg/kg, n = 7–8) for 14 days. A bar magnet (630 mT) was used to generate a magnetic field for drug delivery. The circular top of the magnet was gently placed in contact with the top of the tumor mass and held in place for three hours. This process was repeated every day for 14 days. The tumor volume was calculated on the last injection day using the following formula: (tumor volume) = π/6 × L × W × H (mm^3^) (L; length (mm), W; width (mm), H; height (mm).

### Tail melanoma model with the sliding magnet method

Clone M3 cells (melanoma cells, 3.5 × 10^6^ cells) were grafted by subcutaneous injection into the tails of BALB/c Slc-*nu/nu* mice (6–7 weeks old; 6–10 mice/group) (SLC, Shizuoka, Japan) as previously shown^51^. Three days after grafting, daily single-dose intravenous injections of Fe(Salen) particles (50 mg/kg) were administered into the tail veins of the mice for 14 days. At the time of the initial Fe(Salen) injection, the melanoma appeared as a small area of pigmentation (less than 5 mm in diameter). Preliminary studies demonstrated that mice survived for at least one month after completion of this dosing regimen.

A few minutes after each injection, the circular top of the magnet was gently placed on the area distal to the site of melanoma grafting for 10 minutes. The magnet was then slid 5 mm along the tail and held there for 10 minutes. This sliding and magnet application was repeated over the length of 30 mm for three hours after each injection (see Fig. 7a). After 14 days, the extension of melanoma pigmentation was determined with the NIH Image J program^35^,^36^. This sliding method was chosen because our magnet accumulates Fe(Salen) mostly within 5 mm of its edge (Fig. 3).

The Animal Care and Use Committee at Yokohama City University, School of Medicine, approved all animal studies.

### MR imaging

MR imaging was performed with a 7.0-Tesla horizontal magnet (Jastec, Kobe, Japan with Bruker BioSpin) for *in vitro* sample measurements and with a 9.4-Tesla vertical magnet system (Bruker BioSpin, Ettringen, Germany) for *ex vivo* mouse tail imaging. The *in vitro* samples were measured T1 and T2 relaxation times to determine the relaxivities and to evaluate the concentration dependence on T1- and T2-weighted imaging. Single-slice T1-weighted images were obtained using a spin-echo sequence with the following parameters: repetition time (TR)/echo time (TE) = 400/9.57 ms, matrix size = 256 × 256, field-of-view (FOV) = 60 × 60 mm, slice thickness (ST) = 2 mm, and number of acquisition (NA) = 4. For the T2-weighted imaging and T2 map, single-slice multi-echo imaging were used as follows: TR = 3000 ms, TE = 10–100 ms, number of echoes = 10, matrix size = 256 × 256, FOV = 60 × 60 mm, ST = 2 mm, and NA = 1. For T1 map, single-slice inversion-recovery MRI was performed using a RARE sequence: TR = 10000 ms, effective-TE = 20 ms, RARE factor = 4, inversion time = 52, 100, 200, 400, 800, 1600, 3200, 6400 ms, matrix size = 128 × 128, FOV = 60 × 60 mm^2^, ST = 2 mm, and NA = 1. All data analysis was performed using ParaVision (Bruker-Biospin) and MRVision (Version 1.5, MRVision Co., USA).

For *ex vivo* mouse tail imaging, BALB/c mice (7 weeks old) grafted with melanoma cells (clone M3, 3.5 × 10^6^ cells) were used. Twenty-one days after grafting, the melanomas appeared as large, raised masses with dark pigmentation. After intravenous injection of Fe(Salen) (150 mg/kg) via the tail vein, the edge of the magnet was placed in contact with the side of the melanoma mass for three hours. The tails were then removed, sealed with adhesive to avoid bleeding, and embedded in 1.5% agarose gel. T2*-weighted MRI was immediately obtained using a gradient echo sequence with following parameters; TR/TE = 200/8 ms, flip angle = 30°, ST = 1 mm, FOV = 40 × 40 mm, matrix size = 512 × 512, and NA = 2.

Animal experiments were performed according to the Yokohama City University guidelines for experimental animals. The Animal Care and Use Committee at Yokohama City University, School of Medicine, approved all animal studies.

### Impurity analysis

We conducted elemental analysis, infrared spectral analysis (ATR method), inductively coupled plasma (ICP) mass spectroscopy (Agilent 4500), and X-ray fluorescence analysis (Rigaku, ZSX Primus II) to examine its purity.

### Statistical method

Data are expressed as mean ± SEM. Data were analyzed by one-way ANOVA followed by the Tukey post hoc test using GraphPad Prism software (GraphPad Software, CA, USA). The criterion of statistical significance was set at *p* < 0.05.

The methods section explicitly saying that the methods were carried out in “accordance” with the approved guidelines. For further information please see this guidelines using the following link: http://www.nature.com/srep/policies/index.html#experimental-subjects.

## Author Contributions

H.E. and Y.I. designed the whole study and wrote the manuscript. K.H., M.S. and M.S.Y. conducted X-ray crystallographic study. M.U., R.K., H.F., I.S., M.M.S., T.U. and M.H.S. conducted pharmacological and molecular biological studies. D.J.S. and H.E. conducted the first principle calculation analysis. Y.H., J.H.K. and S.I. conducted chemical studies. J.L. and I.A. conducted MR imaging. M.H.Y. conducted cyclic volutametric study. T.M., N.A. and K.T. conducted magnetic analysis. K.Y. and I.S. conducted transmission electron microscopy (TEM) transmission electron microscopy (TEM) with energy-dispersive X-rays (EDX) studies.

## Supplementary Material

Supplementary Information Movies S1a. Fe(Salen) crystals were attracted by a magnetic needle in aqueous solution, as observed under a microscope.

Supplementary Information Movies S1b Fe(Salen) crystals were attracted by a magnetic needle in aqueous solution, as observed under a microscope.

Supplementary Information Movies S1c. Fe(Salen) crystals were attracted by a magnetic needle in aqueous solution, as observed under a microscope.

Supplementary Information Movie S2. Trapping of Fe(Salen) particles by the magnetic force generated by a permanent magnet in a simulated blood (water) stream.

Supplementary Information Rotating crystal structure of Fe(Salen)

Supplementary Information Supplementary Data

## References

[b1] Ponce A. M. *et al.* Magnetic resonance imaging of temperature-sensitive liposome release: drug dose painting and antitumor effects. J Natl Cancer Inst 99, 53–63 (2007). 17202113 10.1093/jnci/djk005

[b2] Jain R. K. & Stylianopoulos T. Delivering nanomedicine to solid tumors. Nat Rev Clin Oncol 7, 653–664 (2010). 20838415 10.1038/nrclinonc.2010.139 PMC3065247

[b3] Alexiou C. A. *et al.* Locoregional cancer treatment with magnetic drug targeting. Cancer Res 60, 6641–6648 (2000). 11118047

[b4] Namiki Y. *et al.* A novel magnetic crystal-lipid nanostructure for magnetically guided in vivo gene delivery. Nat Nano 4, 598–606 (2009). 10.1038/nnano.2009.202 19734934

[b5] Torchilin V. P. Recent advances with liposomes as pharmaceutical carriers. Nat Rev Drug Discov 4, 145–160 (2005). 15688077 10.1038/nrd1632

[b6] Gupta A. K. & Guputa M. Synthesis and surface engineering of iron oxide nanoparticles for biomedical applications. Biomaterials 26, 3995–4021 (2005). 15626447 10.1016/j.biomaterials.2004.10.012

[b7] Rogers J. A. & Anderson K. E. The potential of liposomes in oral drug delivery. Crit Rev Ther Drug Carrier Syst 15, 421–480 (1998). 9822867

[b8] Calderrazzo C., Floriani R., Henzi R. & L'Eplattenier F. Reactions of some metal carbonyls with chelating compounds containing active protons, especially Schiff's bases. J Chem Soc **A**, 1378–1386 (1969).

[b9] Wells F. V. *et al.* Moessbauer effect and magnetic investigation of the S = 3/2.tautm. S = 1/2 spin crossover in [Fe(salen)NO] and the S = 3/2 state in [Fe(5-salen)NO]. Inorg Chem 21, 2306–2311 (1982).

[b10] Gerloch M., Lewis J., Mabbs F. E. & Richards A. Structure and paramagnetism of five- and six-Co-ordinate N: N bis-salicylidene ethylenediamine iron(III) chloride. Nature 212, 809–810 (1966).

[b11] Routier S. *et al.* DNA cleavage by hydroxy-salicylidene-ethylendiamine-iron complexes. Nucleic Acids Res 27, 4160–4166 (1999). 10518606 10.1093/nar/27.21.4160 PMC148689

[b12] Ansari K. I., Kasiri S., Grant J. D., Woldemariam G. A. & Mandal S. S. Fe(III)-Salen and Salphen Complexes Induce Caspase Activation and Apoptosis in Human Cells. J Biomol Screen 16, 26–35 (2010). 21045212 10.1177/1087057110385227

[b13] Woldemariam G. A. & Mandal S. S. Iron(III)-salen damages DNA and induces apoptosis in human cell via mitochondrial pathway. J Inorg Biochem 102, 740–747 (2008). 18180039 10.1016/j.jinorgbio.2007.11.008

[b14] Pradhan N. *et al.* Induction of apoptosis by Fe(salen)Cl through caspase-dependent pathway specifically in tumor cells. Cell Biol Int 38, 1118–1131 (2014). 24804954 10.1002/cbin.10308

[b15] Drebov N. *et al.* Ab initio screening methodology applied to the search for new permanent magnetic materials. New J Phys 15, 125023 (2013).

[b16] S Honary F. Z. Effect of zeta potential on the properties of nano-drug delivery systems - A review (Part 1). Trop J Pharm Res 12, 265–273 (2013).

[b17] Jonas L., Zellmann E. & Muss W. Elemental Analysis in Electron Microscopy for Medical Diagnostics. Microsc Microanal 13, 222–223 (2007).

[b18] Hergt R. *et al.* Physical limits of hyperthermia using magnetite fine particles. Magnetics, IEEE Transactions on 34, 3745–3754 (1998).

[b19] Rezaee M. A. Elahe., Hunting, Darel. & Sanche, L′eon. DNA-Platinum thin films for use in chemoradiation therapy studies. Bioinorg Chem Appl 2012, 9 (2012). 10.1155/2012/923914 PMC3184495 21977010

[b20] Fukumura H. *et al.* Effect of ascorbic acid on reactive oxygen species production in chemotherapy and hyperthermia in prostate cancer cells. J Physiol Sci 62, 251–257 (2012). 22392350 10.1007/s12576-012-0204-0 PMC10717908

[b21] Minai L., Yeheskely-Hayon D. & Yelin D. High levels of reactive oxygen species in gold nanoparticle-targeted cancer cells following femtosecond pulse irradiation. Sci Rep 3, 2146 (2013). 23828378 10.1038/srep02146 PMC3701901

[b22] Inoue S. & Kawanishi S. Hydroxyl radical production and human DNA damage induced by ferric nitrilotriacetate and hydrogen peroxide. Cancer Res 47, 6522–6527 (1987). 2824034

[b23] De Laat J. & Le T. G. Effects of chloride ions on the iron(III)-catalyzed decomposition of hydrogen peroxide and on the efficiency of the Fenton-like oxidation process. Applied Catalysis B: Environmental 66, 137–146 (2006).

[b24] Manda G., Nechifor M. T. & Neagu T. M. Reactive oxygen species, cancer and anti-cancer therapies. Curr Chem Biol 3, 342–366 (2009).

[b25] Bai J., Rodriguez A. M., Melendez J. A. & Cederbaum A. I. Overexpression of catalase in cytosolic or mitochondrial compartment protects HepG2 cells against oxidative injury. J Biol Chem 274, 26217–26224 (1999). 10473575 10.1074/jbc.274.37.26217

[b26] Edulji S. K. & Nguyen S. T. Catalytic olefin cyclopropanation using μ-Oxo−bis[(salen)iron(III)] complexes. Organometallics 22, 3374–3381 (2003).

[b27] Leung W.-H., Chan E. Y. Y., Chow E. K. F., Williams I. D. & Peng S.-M. Metal complexes of a chiral quadridentate Schiff base. J Chem Soc, Dalton Transactions 7, 1229–1236 (1996).

[b28] Murray K. S. Binuclear oxo-bridged iron(III) complexes. Coord Chem Rev 12, 1–35 (1974).

[b29] Calderazzo F., Floriani C., Henzi R. & L'Eplattenier F. Reactions of some metal carbonyls with chelating compounds containing active protons, especially Schiff's bases. J Chem Soc A: Inorganic, Physical, Theoretical (A), 1378–1386 (1969).

[b30] Khaliullin G. & Okamoto S. Quantum Behavior of orbitals in ferromagnetic titanates: Novel orderings and excitations. Phys Rev Lett 89, 167–201 (2002). 10.1103/PhysRevLett.89.167201 12398749

[b31] Goodenough J. B. Theory of the role of covalence in the perovskite-type manganites [La, M(II)]MnO_3_. Phys Rev 100, 564–573 (1955).

[b32] Chu S. H., Singh D. J., Wang J., Li E. P. & Ong K. P. High optical performance and practicality of active plasmonic devices based on rhombohedral BiFeO_3_. Laser Photo Rev 6, 684–689 (2012).

[b33] Segall M. D. *et al.* First-principles simulation: ideas, illustrations and the CASTEP code. J Phys: Condensed Matter 14, 2717 (2002).

[b34] Koksharov Y. A. Magnetism of nanoparticles: Effects of size, shape, and interactions. In: Gubin, S. P. (ed) Magnetic nanoparticles. Vol.6 (ed. Wiley, Weinheim) (197–254) (2009).

[b35] Yamamoto T., Takiwaki H., Arase S. & Ohshima H. Derivation and clinical application of special imaging by means of digital cameras and Image J freeware for quantification of erythema and pigmentation. Skin Res Technol 14, 26–34 (2008). 18211599 10.1111/j.1600-0846.2007.00256.x

[b36] Green A., Martin N., Pfitzner J., O′Rourke M. & Knight N. Computer image analysis in the diagnosis of melanoma. J Am Acad Dermatol 31, 958–964 (1994). 7962777 10.1016/s0190-9622(94)70264-0

[b37] Lopez-Bergami P. H. *et al.* Rewired ERK-JNK signaling pathways in melanoma. Cancer Cell 11, 447–460 (2007). 17482134 10.1016/j.ccr.2007.03.009 PMC1978100

[b38] Bach D. M., Straseski J. A. & Clarke W. Therapeutic drug monitoring in cancer chemotherapy. Bioanalysis 2, 863–879 (2010). 21083218 10.4155/bio.10.48

[b39] Crawford J. D., Terry M. E. & Rourke G. M. Simplification of drug dosage calculation by application of the surface area principle. Pediatrics 5, 783–790 (1950). 15417279

[b40] Pinkel D. The use of body surface area as a criterion of drug dosage in cancer chemotherapy. Cancer Res 18, 853–856 (1958). 13573353

[b41] Duguet E., Vasseur S., Mornet S. & Devoisselle J. M. Magnetic nanoparticles and their applications in medicine. Nanomed 1, 157–168 (2006). 10.2217/17435889.1.2.157 17716105

[b42] Weissleder R. & Pittet M. J. Imaging in the era of molecular oncology. Nature 452, 580–589 (2008). 18385732 10.1038/nature06917 PMC2708079

[b43] Lan G., Wang Y., Jiang Y., Zhou H. & Yi D. Effects of rare-earth dopants on the thermally grown Al2O3/Ni(Al) interface: the first-principles prediction. J Mater Sci 49, 2640–2646 (2014).

[b44] Hirata K. *et al.* New micro-beam beamline at SPring-8, targeting at protein micro-crystallography. AIP Conf Proc 1234, 863–896 (2010).

[b45] Otwinowski Z. & Minor W. Processing of X-ray diffraction data collected in oscillation mode. in Methods in Enzymology, Vol. 276 (ed. Carter, C. W., Jr.) (307–326) (Academic Press, 1997). 10.1016/S0076-6879(97)76066-X 27754618

[b46] Sheldrick G. M. A short history of SHELX. Acta Crystallogr Section A 64, 112–122 (2008). 18156677 10.1107/S0108767307043930

[b47] Ghose A. K., Viswanadhan V. N. & Wendoloski J. J. A knowledge-based approach in designing combinatorial or medicinal chemistry libraries for drug discovery. 1. A qualitative and quantitative characterization of known drug databases. J Comb Chem 1, 55–68 (1999). 10746014 10.1021/cc9800071

[b48] Marzari N., Vanderbilt D. & Payne M. C. Ensemble density-functional theory for Ab initio molecular dynamics of metals and finite-temperature insulators. Phys Rev Lett 79, 1337–1340 (1997).

[b49] Sato I. *et al.* Hyperthermia generated with ferucarbotran (Resovist®) in an alternating magnetic field enhances cisplatin-induced apoptosis of cultured human oral cancer cells. J Physiol Sci 64(3), 177–83 (2014). 24619404 10.1007/s12576-014-0309-8 PMC10717732

[b50] Wu X., Gong S., Roy-Burman P., Lee P. & Culig Z. Current mouse and cell models in prostate cancer research. Endocr Relat Cancer 20, R155–R170 (2013). 23580590 10.1530/ERC-12-0285 PMC3855867

[b51] Stecca B. *et al.* Melanomas require HEDGEHOG-GLI signaling regulated by interactions between GLI1 and the RAS-MEK/AKT pathways. Proc Natl Acad Sci 104, 5895–5900 (2007). 17392427 10.1073/pnas.0700776104 PMC1838820

