# Supplementary material for: A magnetic anti-cancer compound for magnet-guided delivery and magnetic resonance imaging
Source: Sci Rep. 2015 Mar 17;5:9194. doi: 10.1038/srep09194 (PMC4361848; doi:10.1038/srep09194)
Supplement: Supplementary Information — Supplementary Data [file srep09194-s6.docx]

**Supplementary Data**

**A magnetic anti-cancer compound for magnet-guided delivery and magnetic resonance imaging**

Haruki Eguchi, Masanari Umemura, Reiko Kurotani, Hidenobu Fukumura, Itaru Sato, Jeong-Hwan Kim, Yujiro Hoshino, Jin Lee, Naoyuki Amemiya, Motohiko Sato, Kunio Hirata, David J. Singh, Takatsugu Masuda, Masahiro Yamamoto, Tsutomu Urano, Keiichiro Yoshida, Katsumi Tanigaki, Masaki Yamamoto, Mamoru Sato, Seiichi Inoue, Ichio Aoki and Yoshihiro Ishikawa

**Context of supplementary information**

**1. Supplementary File 1**

**2. Supplementary Figures 1-9**

**3. Supplementary Table 1**

**4. Supplementary Movies 1-3**

**Supplementary File 1**

**First principles calculations**

We used the GGA+U method [^1^](#_ENREF_1) and the CASTEP program package [^2^](#_ENREF_2) using the experimental crystal structure. We calculated the energy difference between the ferromagnetic ordering and two different antiferromagnetic orderings of the Fe spins in the four Fe atom unit cell. We found that ferromagnetism was indeed preferred based on the calculated energies**.**

We then performed PBE GGA calculations using the linearized augmented planewave method (Fig. 5c). We found that Fe is present as a high-spin divalent ion, though with substantial covalency involving the ligands, indicating that this is a hypoligated complex [^3^](#_ENREF_3). This finding is consistent with the cyclic voltammogram (**Fig. S3**). Interestingly, however, the geometrical distance between Fe-O-Fe was relatively short and suggested a trivalent interaction. The apparent difference in valence is most likely due to the nature of the hypoligated complex, which has partly covalent bonding and also unpaired electrons in its structure, as has been reported in various complexes [^3^](#_ENREF_3). Indeed, we found that this iron-salen has a large covalent component through first-principles studies and this was also supported by the structure obtained by X-ray crystallographic analysis. The calculated spin moment of the compound was 4 µ_B_ per Fe atom. This is the expected value for fully polarized high-spin divalent Fe. This high spin moment, together with the covalent bonding, results in an extremely strong spin-dependent hybridization of the Fe 3*d* orbitals with the neighboring ligand (O and N) *p* orbitals. Hybridization with the ligands is stronger in the majority spin channel. This leads to a substantial induced magnetic polarization on the N and O atoms that are bonded to Fe, which is parallel to the Fe moments.

We found substantial induced polarizations at other atoms of the salen moieties that were also parallel to the Fe moments. This finding is similar to the situation in ferromagnetic manganites, such as (La,Ca)MnO_3_ alloys [^4^](#_ENREF_4). Furthermore, we found that the polarization of N and O that are bound to Fe is followed by polarization of C in the salen units. This occurs in a pattern in which there is substantial polarization on every second C but not on the intervening atom, and all of these magnetic polarizations are parallel to the Fe moments. This alternation of atoms with and without moments is a common feature of structures built from *sp*^2^-hybridized C and is also exemplified in long-range ferromagnetic interactions in graphene [^5^](#_ENREF_5). These mathematical analyses indicate that the magnetic moments are distributed over the salen units by the Fe-induced spin-dependent, sequential hybridization of N, O and C and that the magnetic moment is not simply localized at the Fe ions. The details of the intermolecular exchange interactions will depend on the exact structure of the hypoligated Fe(Salen), including the H positions, which must be determined more precisely in future work.

**
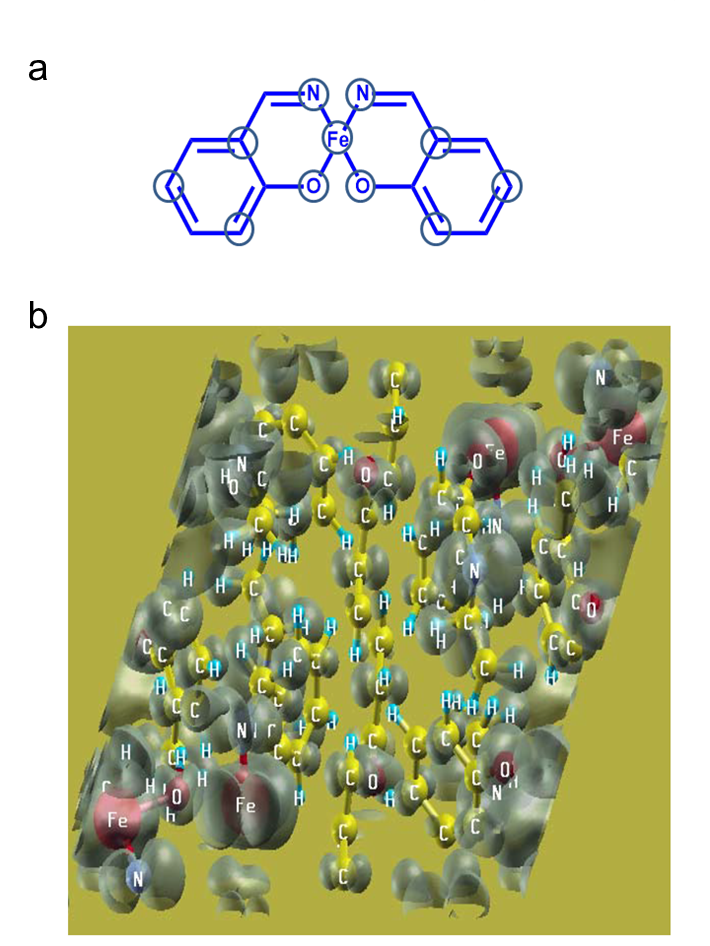
**

**Figure S1 First principles calculations of Fe(Salen)**

In the electronic structure analysis for spin polarization, the spin-polarized density states on *d* orbitals and calculated spin density for one unit cell (4 Fe(Salen) molecules) of the hemihydrate were obtained using the linearized augmented planewave method. The sphere sizes in the augmented planewave calculations were 1.6 bohr (Fe), 1.0 bohr (C, N, and O) and 0.75 bohr (H).

(a) Sites in Fe(Salen) with substantial spin polarization are indicated by circles.

(b) Calculated spin density shown for one unit cell (4 Fe(Salen) molecules) of the hemihydrate. Note the polarization of the N and O neighbors on the Fe and on the π orbitals of alternating C atoms. The spin density contour at 0.001 e/bohr^3^ is shown.

**Mechanism of ROS production and cytotoxicity**

ROS production via Fenton reaction (reaction 1) was also supported by findings in cyclic voltammetry (Figs. S2 and S3); decreased amount of H_2_O_2_ by Fe^2+^(Salen) via Fenton reaction was reflected by decreases in both oxidization and reduction currents.

$\mathrm{Fe}^{2+}+ H_{2} O_{2} \to\mathrm{Fe}^{3+}+OH^{-}+ \cdot OH$ (1)

As shown in Figure S2, cyclic voltammogram of control buffer solution (*black line*) showed mostly zero current. When H_2_O_2_ was added, H_2_O_2_ was readily decomposed at electrode, as shown in below oxidation process (reaction 2) (for detail, see [^6^](#_ENREF_6)), producing a large oxidization current (*A* on *red line)*.

$H_{2}O_{2} \to O_{2}+2H^{+}+2e^{-}$ (2)

This large oxidization current was decreased (*A on blue line*) when Fe^2+^(Salen) was added. This reduction indicates the trivalent status of iron, electrochemically, as previously demonstrated in detail with other iron-salen or iron complexes [^7-9^](#_ENREF_7). Thus, H_2_O_2_ was most likely consumed by Fenton reaction, and the resultant Fe^3+^(Salen) produced this current. This current also mimics that with Fe^2+^ (Salen) alone with the same peak current value (approximately 20 µA) (Fig. S3b).

The consumption and thus decrease of H_2_O_2_ was also reflected by decrease in the reduction current (*B on blue line versus red line*). H_2_O_2_ was decomposed at electrode, producing the reduction current (reaction 3). This reduction current was also decreased because of decreased amount of H_2_O_2_ [^6^](#_ENREF_6).

H_2_O_2_ + e^-^ →　1/2 H_2_ + HO_2_^-^ (3)

We then verified the oxidization of Fe(Salen) and thus its valent state. Ferrocene was used as reference of divalent iron. Cyclic voltammetry demonstrated that the oxidation potential of Fe(Salen) was greater than that of the ferrocene redox potential (Fig. S3), indicating that native Fe(Salen) *per se* was in the divalent state, at least, in its electrochemical behavior. This result also agrees with that of the first principles calculation (Supplementary File 1, *First principles calculations*). This is most likely due to the nature of the hypoligated complex, which has partly covalent bonding and also unpaired electrons in its structure, as reported in various complexes [^3^](#_ENREF_3).


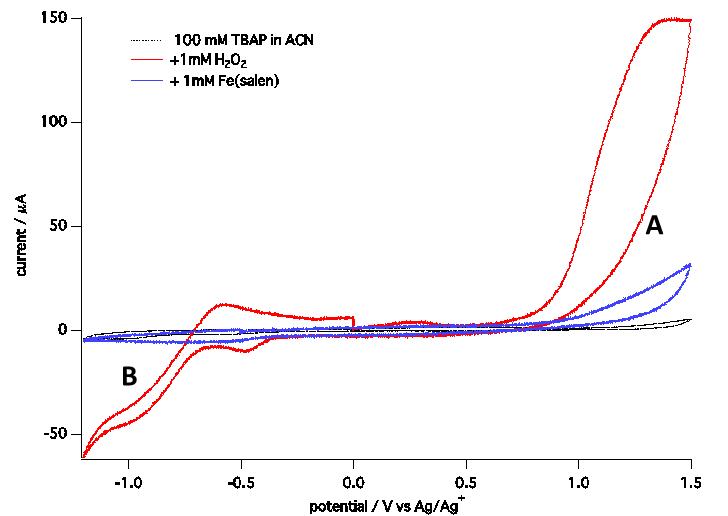


**Figure S2 Cyclic voltammogram of Fe^2+^(Salen)**

Cyclic voltammogram of (a) 100 mM TBAP in ACN (*black line*), (b) 1 mM H_2_O_2_ in 100 mM TBAP in ACN (*red line*), (c) 1 mM iron(II)-salen・0.5H_2_O in 1 mM H_2_O_2_ and 100mM TBAP in ACN (*blue line*) with scan rate of 50 mV s^-1^ are shown. Cyclic voltammogram of 100mM TBAP in ACN as a control buffer solution (*black line*) showed mostly zero current with respect to the applied voltage between -1.2 and 1.5 V vs Ag/Ag^+^. When H_2_O_2_ was added, H_2_O_2_ was decomposed and the oxidization current (A) was detected (*red line)*. The oxidization current was approximately 140 $\mu A$ at 1.2 V vs Ag/Ag^+^. When Fe^2+^(Salen) was then added, the oxidization current was decreased on *blue line*. Of note this current mimics that with Fe^2+^(Salen) alone (Fig. S3b with different scale axis). This decrease was most likely due to the consumption of H_2_O_2_ by Fe^2+^ via Fenton reaction. In the same way, the reduction of H_2_O_2_ at the potential below -0.6 V vs Ag/Ag^+^ was observed (*B on* *red line*), and this reduction current (B) was also decreased on *blue line*.

**Figure S3 Cyclic voltammogram of Fe(salen)**

(a) Cyclic voltammogram of 1 mM ferrocene (Fe(C_5_H_5_)_2_) as the reference in 100 mM TBAP with a scan rate of 0.05 V s^-1^. The voltammogram of ferrocene showed a symmetrical form with the midpoint potential at 0.1 V versus Ag/Ag^+^, indicating that this was the redox potential for ferrocene. Ferrocene was readily oxidized (upward peak) and then reduced (downward peak), indicating that the redox reaction occurred in a reversible manner.

(b) Cyclic voltammogram of 1.4 mM Fe(Salen) in 100 mM TBAP acetonitrile solution with a single scan rate of 0.05 V s^-1^. Native Fe(Salen) was examined in solution, and the cyclic voltammogram showed a non-symmetrical form. The start potential was 0 V versus Ag/Ag^+^ and swept +1.5 V, returned to -0.5 V and then back to 0 V. Thus, Fe(Salen) was oxidized at the upward line at +0.5 V versus Ag/Ag^+^. Because this oxidation potential of Fe(Salen) (0.5V) was greater than the ferrocene redox potential (a), Fe(Salen) was in the divalent state at the start potential of 0V. Note that the oxidation peak, i.e., the upward peak of Fe(Salen), was much broader than that of ferrocene, indicating that the Fe(Salen) was oxidized in a different manner from ferrocene. Because the reduction peak (or downward peak) was not observed with Fe(Salen), Fe(Salen) was most likely decomposed after oxidization.

**Mechanism of drug delivery and cytotoxic effect**.

Putting all together with the above findings, Fe(Salen) is attracted by magnet and exhibit cytotoxicity. In the form of nanoparticles, which are relatively large and not disturbed by thermal variation, they are attracted by magnet. When such nanoparticles are destroyed or incubated for long time period and at high temperature, small particles or single molecules of Fe(Salen) are eluted. These eluted Fe(Salen) molecules can no longer be attracted by magnet because they are too small, however, they can reacts with H_2_O_2_, which is released from cancer cells, to produce ROS via Fenton reaction. This ROS exhibits cytotoxicity. Importantly, the production of H_2_O_2_ is greater in cancer cells than in normal cells [^10-12^](#_ENREF_10), implicating greater cytotoxicity of Fe(Salen) on cancer cells.

**
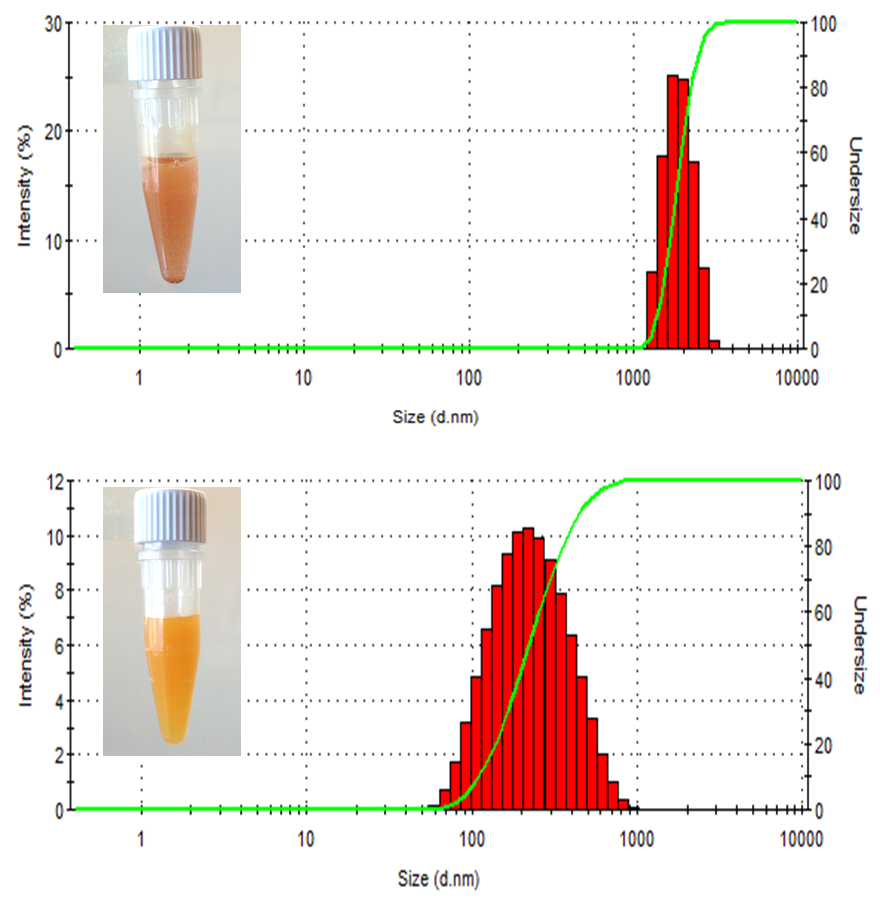
a**

**b**

**Figure S4 The size distribution of the unsonicated and sonicated Fe(Salen) particles suspensions**

Dynamic light scattering histogram showing the size distribution of the unsonicated (a) and sonicated (b) Fe(Salen) particles suspensions. Fe(Salen) particles have an average diameter consistent with TEM. The photographs of Fe(Salen) particles solutions are also shown (inset).

**a**


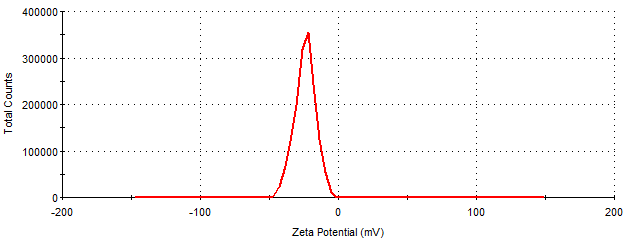

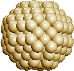

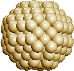

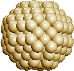

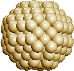

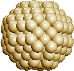

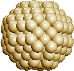

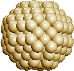


**
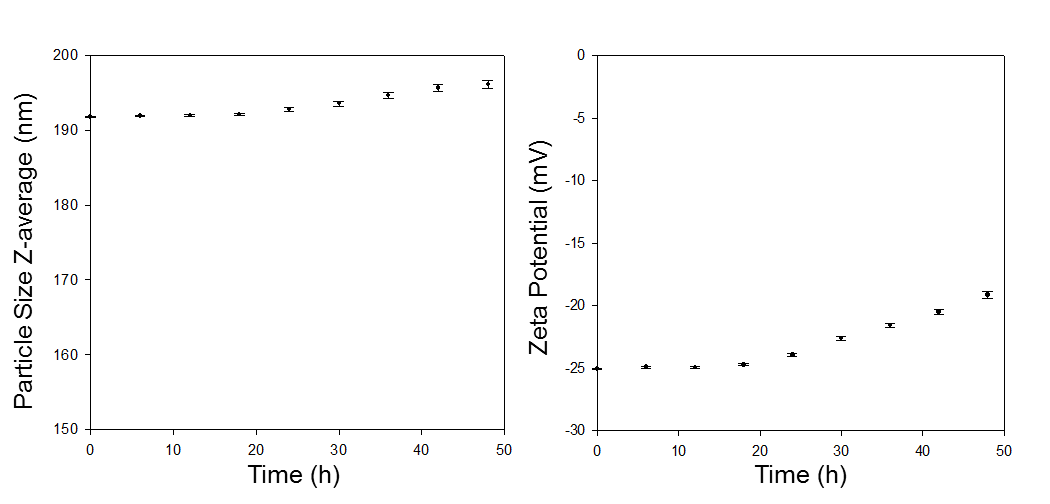
**

**b**

**Figure S5 The zeta potential value of Fe(Salen) particles**

The stability of the colloidal suspension of sonicated Fe(Salen) particles was also determined by zeta potential measurements. The Fe(Salen) particles showed a zeta potential value of -24.1 mV, indicating a stable colloidal dispersion in PBS buffer solution [^13^](#_ENREF_13). The schematic represents a molecular model of a Fe(Salen) particle structure in the presence of PBS ions (a). The DLS and zeta potential measurements as a function of time to evaluate the colloidal stability of Fe(Salen) particles (b).

**
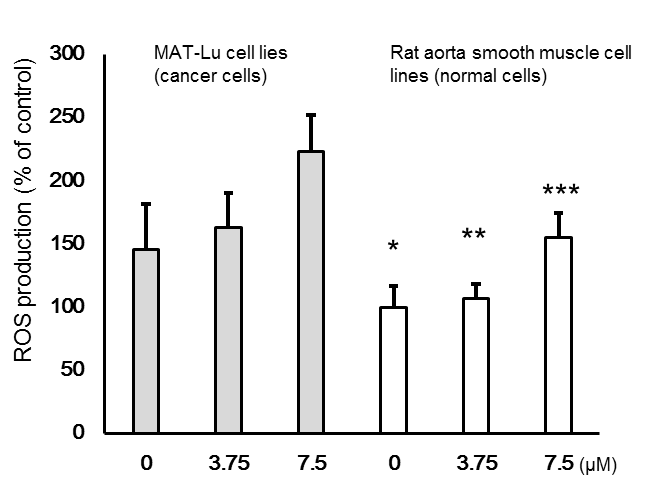
**

**Figure S6 Fe(Salen) particles - induced ROS generation in comparison with cancer cells and normal cells**

Effect of sonication of Fe(Salen) particles on ROS generation in MAT-Lu cells (cancer cells) and rat aorta smooth muscle cells (normal cells). ROS generation was measured in the presence of sonicated Fe(Salen) particles using the fluorescent dye 2',7'-dichlorofluorescein diacetate (DCFH-DA) This result demonstrated that ROS generation effect of Fe(salen) might be greater on cancer cells than normal cells (n=6, **p*<0.05, ***p*<0.01, ****p*<0.001 vs. cancer cells in each concentration).

**a**

**
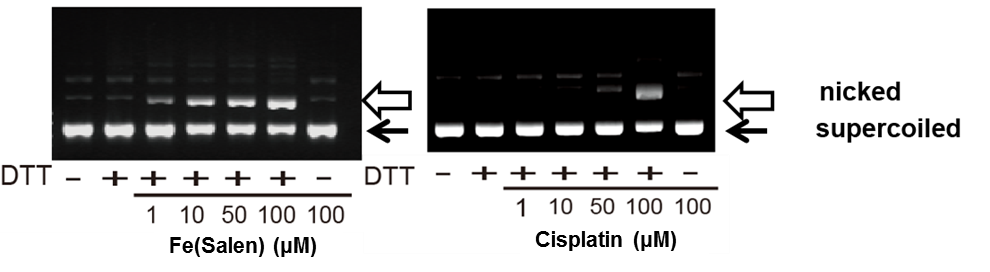
**

**
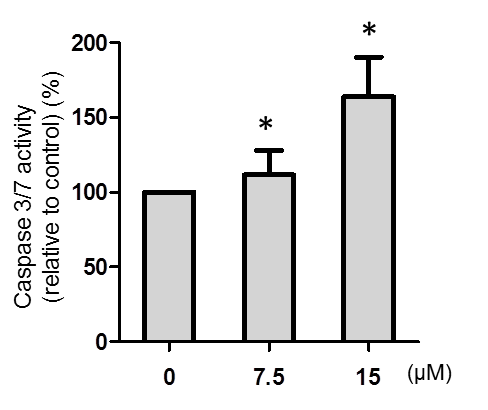
b**

**Figure S7 Fe(Salen) particles induces DNA nicking and activates caspase 3/7**

(a) Representative electrophoresis of purified supercoiled plasmid DNA in the presence of various concentrations of Fe(Salen) or cisplatin. White arrows indicate nicked DNA and black arrows indicate supercoiled DNA. DNA nicking was similarly induced (left). Such DNA nicking was similar to that of cisplatin (right).

(b) Fe(Salen) induced caspase 3/7 activation in MST-Lu cells. (n=3, **p*<0.05 vs. control).

**
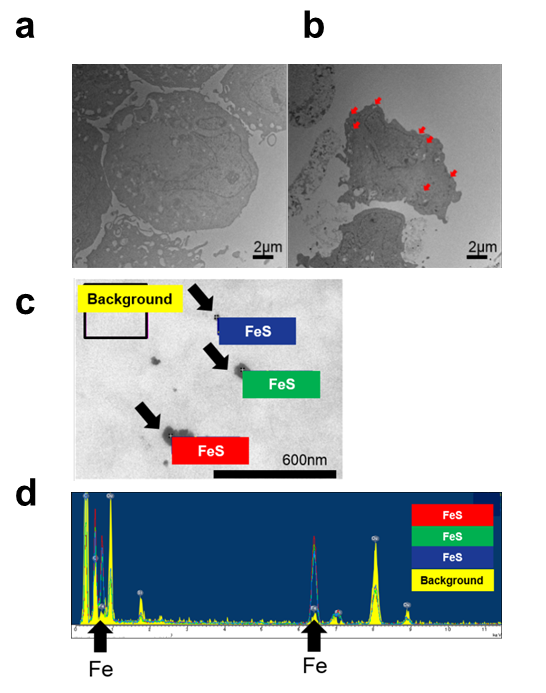
**

**Figure S8 Cellular uptake of Fe(Salen) particles**

Cellular uptake of Fe(Salen) particles was analyzed by transmission electron microscopy (TEM) and energy-dispersive X-rays (EDX) in VX2cell. (**a**) TEM of control cells, (**b**) TEM of cells after incubation with 30 μM Fe(Salen) particles. A calibration bar (2 µm) is shown. Note that cells were shrunk and vacuolated, proceeding to apoptotic cellular death with Fe(Salen) particles. (**c**) Fe(Salen) particles in high-power field of TEM. Arrows indicate Fe(Salen) particles, which were analyzed by EDX in (d). The calibration bar (600 nm) is shown. (**d**) Spectrum analysis of Fe(Salen) particles by EDX. Arrows indicate specific peak spectrum for iron. Note that red, green, and blue lines for Fe(Salen) particles in (c) showed the peak spectrum of iron while yellow line for background did not.

**
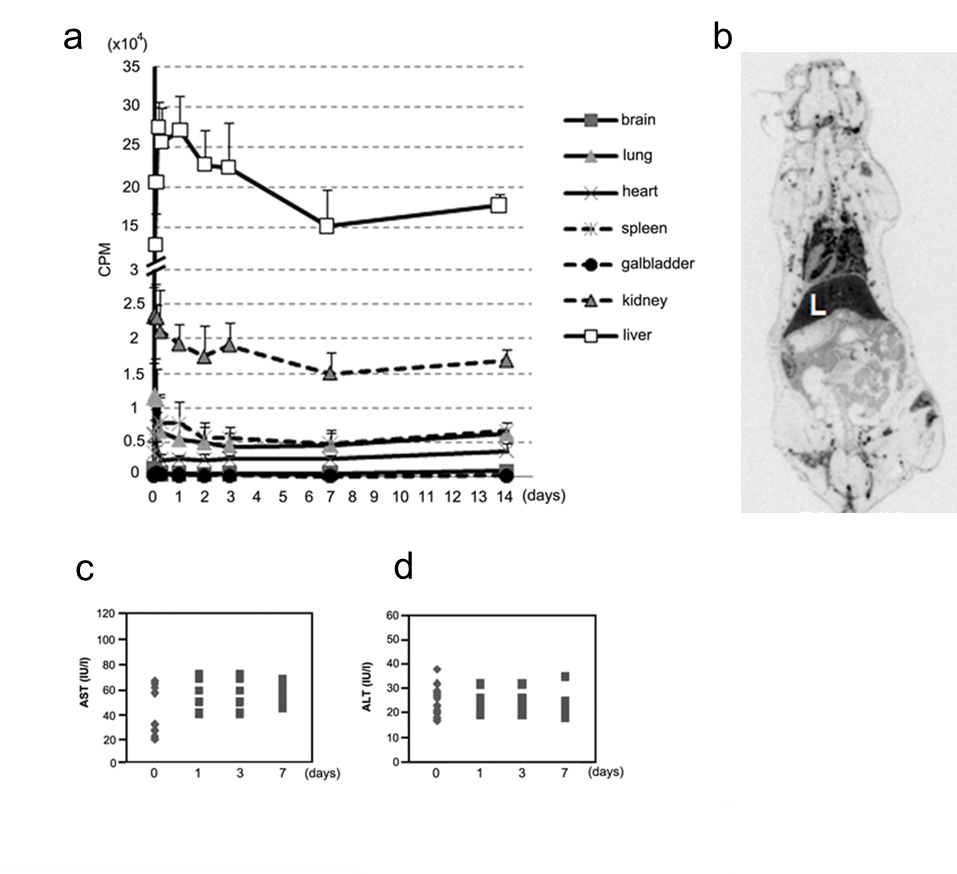
**

**Figure S9 Fe(Salen) tissue distribution, metabolism and toxicity**

(a) Tissue distribution of Fe(Salen) .Changes in tissue content (cpm of each tissue) are shown following a single injection of [^59^Fe]- Fe(Salen) (50 mg/kg).The LD_50_ value was approximately 190 mg/kg (150 to 337.5 mg/kg) as determined by up-down test. (b) Representative autoradiography of tissue distribution (50 mg/kg in normal mouse). Note that Fe(Salen) was mostly metabolized throughout liver-gallbladder. (c and d). Effect of Fe(Salen) on hepatic function. Changes in serum hepatic enzymes (AST (c) and ALT (d)) are shown following a single Fe(Salen) injection (50 mg/kg).

**
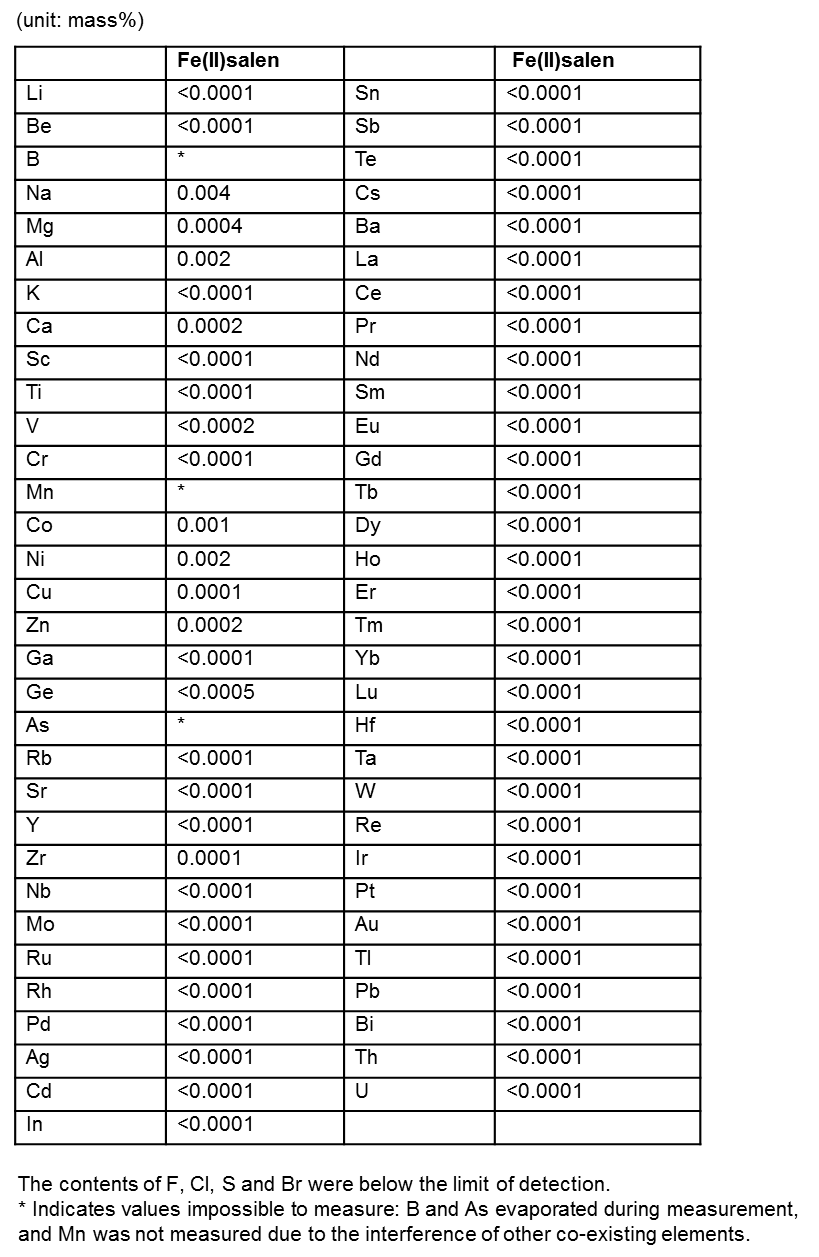
**

**Table S1 Impurity analysis**

The analysis of this commercially obtained Fe(Salen) particles gave values close to the expected ones: C (58.21 vs. 57.73), H (4.27 vs. 4.42), Fe (16.92 vs. 17.2), N (8.49 vs. 8.49). We also performed additional analyses. On infrared spectra analysis (ATR method; JASCO FT/IR 4100 spectrometer), Fe(Salen) particles showed the following IR bands (neat): 1619, 1384, 1336, 1302 cm^-1^. Inductively coupled plasma (ICP) mass spectroscopy (Agilent 4500) and X-ray fluorescence analysis (Rigaku, ZSX Primus II) did not detect the presence of any metal, except for iron, that might account for magnetism in the Fe(Salen) samples.

**Movies S1a, b, and c. Fe(Salen) crystals were attracted by a magnetic needle in aqueous solution, as observed under a microscope.**

Videos were acquired at different concentrations of Fe(Salen) particles (**a**, 0.6 mg/ml; **b**, 1 mg/ml; and **c**, 3 mg/ml). Note that magnetic attraction was independent of the shapes and sizes of particles.

**Movie S2. Trapping of Fe(Salen) particles by the magnetic force generated by a permanent magnet in a simulated blood (water) stream.**

The photo in **Figure 1a** and this movie were recorded under the same conditions.

**Movie S3. Rotating crystal structure of Fe(Salen)**

The rotating crystal structure of Fe(Salen) is shown so that the Fe-O-Fe angle (146.359 degrees) can be clearly seen. Green, red, blue, brown, and white atoms represent carbon, oxygen, nitrogen, iron and hydrogen, respectively.

**Supplementary Materials and Methods**

**Dynamic light scattering (DLS)**

The hydrodynamic size and colloidal stability of the Fe(Salen) particles were determined by dynamic light scattering (DLS) and zeta potential analysis using a Zetasizer Nano ZSP (Malvern Instruments, Ltd.). Both DLS and zeta potential data were obtained from twelve runs per measurement and exhibited a uniform distribution between runs, corresponding to a high quality criterion.

**Cyclic voltammetry**

Cyclic voltammetry was carried out to characterize the valence of iron-salen in a solution [^7^](#_ENREF_7). It was also used for the valence state of other iron complexes [^8^](#_ENREF_8)^,^[^14^](#_ENREF_14). We carried out the cyclic voltammetry with a conventional three electrode configuration; platinum was used as the working and counter electrode and Ag/Ag^+^ (10 mM AgNO_3_ and 100 mM TBAP (tetra-butyl ammonium perchlorate acetonitrile solution)) was used as the reference electrode (commercially available, RE-7 Non Aqueous reference electrode, BAS Inc., Tokyo, JAPAN). The cyclic voltammetry was started 30 minutes after preparing a solution of 1.4 mM iron(II)-salen in 100 mM TBAP with a scan rate of 0.05 V s^-1^ . For a reference, we measured the cyclic voltammogram of 1 mM ferrocene (Fe(C_5_H_5_)_2_) in 100 mM TBAP with a scan rate of 0.05 V s^-1^.

**Electrophoresis of nicked plasmid DNA**

To evaluate the DNA cleavage effect of Fe(Salen) and cisplatin, we performed electrophoresis of purified plasmid DNA [^15^](#_ENREF_15). Supercoiled plasmid DNA (pcDNA3), Fe(II)salen or cisplatin, and 1 mM dithiothreitol (DTT) were mixed and incubated at 37°C for 10 minutes. The mixture was then electrophoresed using 0.8 % agarose gel containing 0.1 ng/ml ethidium bromide at 100 V for 30 minutes. The bands were visualized by Dolphin-View (Kurashiki Kobo, Osaka, Japan).

**Caspase 3/7 assay**

Caspse 3/7 activity assay was performed using the SensoLyte Homogeneous AMC Caspsse 3/7 Assay Kit (AnaSpec, Fremont, CA) as described previously [^16^](#_ENREF_16).

**Fe(Salen) particles absorption, distribution, metabolism, excretion and toxicity studies**

[^59^Fe]-Fe(Salen) particles (50 mg/kg) was administered intravenously into C57BL/6 male mice (7-8 wk old), followed by harvesting and dissection of various organs (brain, lung, heart, liver, spleen, kidney, small intestine, colon, gall bladder and testis) at indicated time points (0-14 days). The gamma ray per gram of each organ was measured using a 3” 1480 Automatic Gamma Counter Wizard^TM^. Similarly, liver enzymes, i.e., aspartate aminotransferase (AST) and alanine aminotransferase (ALT), were measured in serum at 1, 3 and 7 day after injection. LD_50_ values were determined by the up-down test in BALB/cClSlc mice (7 wk old, male).

**Supplementary References**

1. Chu, S.H., Singh, D.J., Wang, J., Li, E.P. & Ong, K.P. High optical performance and practicality of active plasmonic devices based on rhombohedral BiFeO3. *Laser Photo Rev* **6**, 684-689 (2012).

2. Segall, M.D.*, et al.* First-principles simulation: ideas, illustrations and the CASTEP code. *J Phys: Condensed Matter* **14**, 2717 (2002).

3. Pauling, L. The nature of the chemical bond and the structure of molecules and crystals: an introduction to modern structural chemistry. *Cornell University Press, Ithaca* (1960).

4. Pickett, W.E. & Singh, D.J. Electronic structure and half-metallic transport in the La1-_X_CaxMnO_3_ system. *Phys Rev B* **53**, 1146-1160 (1996).

5. Pisani, L., Montanari, B. & Harrison, N.M. A defective graphene phase predicted to be a room temperature ferromagnetic semiconductor. *New J Phys* **10**, 033002 (2008).

6. Cofre, P. & Sawyer, D.T. Redox chemistry of hydrogen peroxide in anhydrous acetonitrile. *Inorg Chem* **25**, 2089-2092 (1986).

7. Bard, A.J. & Faulkner, L.R. (eds.). *Electrochemical Methods: Fundamentals and Applications* (Wiley, 2000).

8. Bechtold, T. & Turcanu, A. Iron-complexes of bis(2-hydroxyethyl)-amino-compounds as mediators for the indirect reduction of dispersed vat dyes- cyclic voltammetry and spectroelectrochemical experiments. *J Electroanal Chem* **591**, 118-126 (2006).

9. Hall, D. & Chu, S.Y.F. Cyclic voltammetry of cryptands and cryptates containing the ferrocene unit. *J Organometallic Chem* **498**, 221-228 (1995).

10. Laurent, A.*, et al.* Controlling tumor growth by modulating endogenous production of reactive oxygen species. *Cancer Res* **65**, 948-956 (2005).

11. Chan, S.W.*, et al.* Mitochondrial DNA damage is sensitive to exogenous H_2_O_2_ but independent of cellular ROS production in prostate cancer cells. *Mutat Res Fund Mol Mech Mut* **716**, 40-50 (2011).

12. Han, Y. & Chen, J.Z. Oxidative stress induces mitochondrial DNA damage and cytotoxicity through independent mechanisms in human cancer cells. *BioMed Res Int* **2013**, 8 (2013).

13. S Honary, F.Z. Effect of Zeta Potential on the properties of nano-drug delivery systems - A review (Part 1). *Trop J Pharm Res* **12**, 265-273 (2013).

14. Otwinowski, Z. & Minor, W. Processing of X-ray diffraction data collected in oscillation mode. *Methods in Enzymology*, Vol. 276 (ed. Charles W. Carter, Jr.) (307-326) (Academic Press, 1997).

15. Nakao K, O.Y. *et al*. The synergistic effects of hyperthermia and anticancer drugs on induction of apoptosis. *Med Electron Microsc* **33**, 44-50 (2000).

16. Ansari, K.I., Kasiri, S., Grant, J.D., Woldemariam, G.A. & Mandal, S.S. Fe(III)-Salen and salphen complexes induce caspase activation and apoptosis in human cells. *J Biomol Screen* **16**, 26-35 (2010).
